# Bezafibrate treatment rescues neurodevelopmental and neurodegenerative defects in 3D cortical organoid model of MAPT frontotemporal dementia

**DOI:** 10.1002/alz.70419

**Published:** 2025-08-14

**Authors:** Federica Cordella, Lorenza Mautone, Debora Salerno, Lucrezia Tondo, Silvia Ghirga, Chiara D'Antoni, Erika Parente, Maria Anele Romeo, Mara Cirone, Paola Bezzi, Silvia Di Angelantonio

**Affiliations:** ^1^ Department of Physiology and Pharmacology “V. Erspamer” and Center for Research in Neurobiology “Daniel Bovet” Sapienza University of Rome Roma Italy; ^2^ Center for Life Nano‐ & Neuro‐Science Istituto Italiano di Tecnologia Roma Italy; ^3^ Department of Molecular Medicine Sapienza University of Rome Roma Italy; ^4^ Department of Experimental Medicine Sapienza University of Rome Roma Italy; ^5^ Department of Fundamental Neurosciences University of Lausanne Lausanne Switzerland; ^6^ D‐Tails Research srl BC Rome Italy

**Keywords:** calcium imaging, cortical organoids, mitochondria, neurodegeneration, neurodevelopment, synapses, tau, tauopathies, transcriptomics

## Abstract

**INTRODUCTION:**

The intronic MAPT mutation IVS10+16 is linked to familial frontotemporal dementia, causing hyperphosphorylation and accumulation of tau protein, resulting in synaptic and neuronal loss and neuroinflammation in patients. This mutation disrupts MAPT gene splicing, increasing exon 10 inclusion and leading to an imbalance of 3R and 4R Tau isoforms.

**METHODS:**

We generated patterned cortical organoids from isogenic control and mutant human induced pluripotent stem cell (iPSC) lines. Nanostring gene expression analysis, immunofluorescence, and calcium imaging recordings were used to study the impact of the MAPT IVS10+16 mutation on neuronal development and function.

**RESULTS:**

Tau mutant cortical organoids showed altered mitochondrial function and gene expression related to neuronal development, with synaptic markers and neuronal activity reduction. Bezafibrate treatment restored mitochondrial content and rescued synaptic functionality and tau physiology.

**DISCUSSION:**

These findings suggest that targeting mitochondrial function with bezafibrate could potentially reverse tau‐induced neurodevelopmental deficits, highlighting its therapeutic potential for tauopathies like frontotemporal dementia.

**Highlights:**

- The IVS 10+16 MAPT mutation significantly disrupts cortical differentiation and synaptic maturation, evidenced by downregulated genes associated with synapses and neuronal development.
- Tau‐mutant cortical organoids exhibit mitochondrial dysfunction, with fewer and smaller mitochondria alongside tau hyperphosphorylation and aggregation, which further contribute to neuronal damage and disease progression.
- Treatment with bezafibrate effectively normalizes mitochondrial parameters, enhances neuronal integrity and synaptic maturation, and restores network functionality, showcasing its promise as a therapeutic strategy for tauopathies.
- The 3D in vitro disease model used in this study proves valuable for studying tauopathies and testing new drugs, effectively mimicking key aspects of tau‐related neurodegeneration.

## 1 BACKGROUND

Frontotemporal dementia (FTD) is a debilitating neurodegenerative disorder characterized by the degeneration of neuronal cells within the frontal and temporal lobes of the brain, primarily affecting personality, behavior, language, and executive functioning.^1^ Considering the diverse spectrum of pathological mechanisms contributing to FTD, abnormal changes in tau protein represent a critical avenue of investigation. Tau, encoded by the MAPT gene, is a multitasking protein, and among its functions interacts with tubulin, enhancing its polymerization into microtubules, and regulates their assembly, dynamics, and spatial organization.^2^, ^3^ The alternative splicing of tau pre‐mRNA is developmentally regulated, resulting in the production of six isoforms that differ in containing three (3R) or four (4R) microtubule‐binding regions in the carboxyl terminal and one (1N), two (2N), or zero (0N) amino terminal inserts.^4^

Among tau mutations linked to FTD, the MAPT intronic IVS 10+16 mutation, located within the MAPT gene on chromosome 17, has gained significant attention due to its association with a familial form of the disease.^5^ This mutation enhances the probability of the inclusion of exon 10 within tau transcripts, resulting in the production of tau isoforms with altered microtubule‐binding properties and a propensity for pathological aggregation,^6^ with an increase in 4R tau isoform splicing, leading to significant alterations in neuronal functionality^6^, ^7^ and retinal development.^8^

Among the roles of tau in shaping neuronal homeostasis, its interaction with mitochondria has been highlighted in physiological and pathological conditions as FTD.^2^, ^9^, ^10^, ^11^, ^12^, ^13^ Several studies suggest a potential feedback loop where tau dysregulation impairs mitochondrial homeostasis, and mitochondrial dysfunction exacerbates tau pathology by increasing oxidative stress and hindering the cellular clearance of abnormal tau aggregates. This mitochondria‐tau crosstalk may play a crucial role in triggering and worsening diseases such as Alzheimer's disease (AD) and FTD.^13^, ^14^

Given the early onset and poor prognosis of FTD, there is an urgent need to develop innovative in vitro models that more accurately recapitulate the disease's key features. These models are essential for dissecting molecular pathological mechanisms, identifying novel therapeutic targets, and testing novel and repurposed drug candidates. Over the years, transgenic animal models have been instrumental in elucidating FTD‐associated genetic mutations and the resultant functional changes that characterize this pathology. Although mouse models can replicate key pathological hallmarks such as neurofibrillary tangle deposition, they do not fully reproduce the pathological phenotypes observed in humans, particularly in terms of neuronal loss and neurodegeneration.^15^, ^16^ Therefore, induced pluripotent stem cell (iPSC)‐derived cortical organoids, characterized by 3D structure, represent a more suitable in vitro tool for studying pathological events that occur in vivo.^17^ Notably, the brain organoid model recapitulates the early stages of human cortical development, allowing the evaluation of phenotypic perturbations in neuro‐glia development during critical developmental periods that are otherwise challenging to investigate in humans, thus giving the opportunity to underscore neurodevelopmental deficits associated to typical neurodegenerative disorders.^18^

In this study, we developed a human iPSC‐derived cortical organoid model based on control and isogenic human iPSC harboring the intronic MAPT IVS 10+16 mutation. Our findings, by molecular barcoding approach, are important to identify molecular signatures in neurological diseases and degenerative disorders, indicating that the intronic MAPT IVS10+16 mutation leads to delayed and impaired cortical development due to reduced mitochondrial functions, affecting the maturation and function of both neuronal and glial cells. This mutation also results in the increased expression of 4R tau isoforms and in the accumulation of pathological tau forms. Additionally, we investigated the potential effects of bezafibrate (BZ), agonist of the master regulator of mitochondrial biogenesis PGC1α in our in vitro disease model. Our results show that BZ treatment restores neuronal cytoskeletal morphology and mitochondrial function, promotes neuronal maturation, and rescues synaptic activity, reducing tau hyperphosphorylation, demonstrating a promising therapeutic potential for tauopathies.

## 2 METHODS

### 2.1 Maintenance of human iPSC

iPSC0028 (EBiSC – Sigma) and isogenic SIGI001‐A‐13 (IVS10+16) (EBiSC – Sigma) hiPSC lines were cultured in mTeSR PLUS medium (STEMCELL technologies) and hESC‐qualified Matrigel (CORNING) functionalized plates.^19^, ^20^, ^21^, ^22^ The culture medium was refreshed every other day, and cells were passaged with non‐enzymatic methods every 4–5 days. Cells were routinely tested for mycoplasma contamination.^23^, ^24^

### 2.2 Generation of cortical organoids

Cortical organoids had been generated through modification of an established protocol.^25^ Briefly, hiPSCs were treated with Accutase (Innovate Cell Technologies, AT‐104) at 37°C for 4–7 min and dissociated into single cells. To obtain uniformly sized spheroids, AggreWell800 (STEMCELL technologies) containing 1800 microwells were used (800 µm/each). Approximately 2 × 10^6^ single cells were added per AggreWell800 well in mTeSR PLUS medium (STEMCELL technologies) supplemented with 20 µM ROCK inhibitor Y‐27632 (STEMCELL technologies), centrifuged the plate at 100 × g for 3 min to capture the cells in the microwells, and incubated at 37°C with 5% CO_2_. The seeding day is defined as day 0 (D0). After 24 h (D1), half of the medium was replaced with neural induction medium (NIM) composed of DMEM/F‐12 (1:1) (Sigma Aldrich) supplemented with 1× Sodium pyruvate (GIBCO), 1× B27 w/oA (GIBCO) 1× GlutaMAX (GIBCO), 1× N2 supplement (Thermo Fisher Scientific), and 1× MEM non‐essential amino acids (NEAA) (GIBCO). NIM was always freshly supplemented with 1 µg/ml heparin (Sigma Aldrich), 1x SB 431542 (1000×; final 10 µM) (Biogems), 1× DORSO (400x; final 2.5 µM) (Sigma Aldrich), 1× XAV 939 (4000×; final 2.5 µM). All the small molecules, including heparin, were added from D1 to D8, and the medium was changed every day. At D8, organoids were harvested by firmly pipetting (with a cut end of a P1000 tip) medium in the well up and down and transferred into 60 mm ultra‐low attachment plastic dishes (Corning, 3262). The spheroids were resuspended in 5 mL of neural differentiation medium (NDM) containing Neurobasal basic (Thermo Fisher Scientific), 1× B‐27 w/oA, 1× GlutaMax, and kept in floating condition by shaking the cultures on an orbital shaker (40–60 RMP). The NDM was supplemented with 20 ng/ml fibroblast growth factor 2 (FGF2; R&D Systems) and 1 µg/ml heparin, with medium change every other day. At around D30, to promote differentiation of the neural progenitors into neurons, FGF2 and heparin were replaced with 20 ng/ml brain‐derived neurotrophic factor (BDNF; Peprotech), 20 ng/ml GDNF (Peprotech), and 20 ng/ml ascorbic acid. The medium was changed every 3 days. From D50 to D100 NDM was modified by replacing the Neurobasal basic with Neurobasal A. At this point, only 20 ng/ml BDNF (Peprotech) was freshly added, and the medium was changed every 4 days until further characterization. BZ (B7273; Merck) treatment was performed by adding 5 µM BZ to the culture medium from D70 to D100 (Figure S1).

**RESEARCH IN CONTEXT**

1. **Systematic review**: We searched PubMed, Google Scholar, and Web of Science for studies on the MAPT IVS10+16 mutation's impact on tauopathies, focusing on neuronal development, synaptic function, and mitochondrial involvement. Key terms included “MAPT IVS10+16 mutation,” “tauopathy,” “neuronal development,” “synaptic function,” and “mitochondrial function.”
2. **Interpretation**: Our findings reveal that the MAPT IVS10+16 mutation disrupts mitochondrial function, altering gene expression related to neuronal development and synaptic structures, impairing neuronal and glial maturation. Bezafibrate treatment restored mitochondrial content, synaptic functionality, and tau physiology in mutant‐derived cortical organoids, suggesting it as a potential therapeutic strategy for tauopathies.
3. **Future directions**: Future research should investigate the molecular mechanisms underlying the bezafibrate's therapeutic effect and its long‐term efficacy and safety in vivo, in humanized mouse models. Additionally, the possibility to combine bezafibrate with other therapeutic agents used to treat tauopathies will be worth assessing.

### 2.3 Dissociation of cortical neurons from cortical organoids

To obtain 2D cortical cultures, we followed a published protocol.^26^ Up to five brain organoids at D70 were collected and treated with Accutase (Innovate Cell Technologies, AT‐104) at 37°C for 4–7 min and gently dissociated into single cells. Cells were then centrifuged at 300 g for 5 min and resuspended in 2 mL of NDM‐A medium supplemented with 20 ng/ml BDNF (Peprotech) and plated onto poly‐L‐ornithine/laminin‐coated dishes at a density of 80.000 cells per cm^2^. The medium was replaced every other day (Figure S1).

### 2.4 Immunostaining, image acquisition, and analysis of neuronal and astrocytic markers

Cortical organoids were collected at D30 and D100, incubated in 4% paraformaldehyde (PFA; Sigma Aldrich) solution for 2 h, and then placed in phosphate buffered saline (PBS) with 30% sucrose overnight at 4 °C. The following day, the sucrose solution was removed, and organoids were placed on OCT (at least three organoids on each OCT drop) and cryosectioned using a standard cryostat (Leica CM1860, Leica Biosystems). 20 µm thick sections were collected on Ultra Plus slides (Thermo Fisher Scientific) and stored at 4 °C. For immunostaining, organoid sections were quickly washed for 10 min in PBS and then incubated for 40 min with a warm antigen retrieval solution (95°C) containing 1× citrate buffer, at pH 6.0 (Sigma Aldrich). After a double washing of 5 min in PBS with Tween‐20 (0.1%), sections were blocked in 0.3% Triton‐X and 5% goat serum, 1% bovine serum albumin (BSA), and 200 mM glycine (Sigma Aldrich) in PBS for 1 h. Organoid slices were then incubated overnight with primary antibodies in 0.3% Triton‐X and 5% goat serum in PBS as reported in Table S1. The day after, the primary antibody solution was washed out, and slices were incubated with secondary antibodies for 2 h at room temperature in a dark room. AlexaFluor secondary antibodies (Thermo Fisher Scientific) were used at the concentration of 1:500 (Table S2), and Hoechst (Sigma Aldrich, 1:500) was used for nuclei staining. Finally, after three washes with 0.1% Tween‐20 in PBS, organoid sections were mounted with ProLong Diamond Antifade Mountant (Thermo Fisher Scientific) and sealed with nail polish. The slides were stored at 4 °C until acquisition. Images were acquired with an inverted microscope Olympus IX73 equipped with the X‐Light V3 Spinning Disk Confocal module (Crest Optics), LDI laser source and a Prime BSI Scientific CMOS (sCMOS) 6.5 µm pixels (Photometric). All the images were acquired with a 60x/ NA 1.42 and 100x/NA 1.45 oil objectives (Olympus) in stack with z‐step of 0.2 µm exploiting the Metamorph software version 7.10.2 (Molecular devices, Wokingham, UK).

Fluorescence images of PAX6, CTIP2, TBR1, and glial fibrillary acidic protein (GFAP) staining (images acquired with a 60× objective) were analyzed through a custom code developed in MATLAB environment.^27^ Mean Z‐projections over 20 planes of the image stacks were subjected to a pre‐processing step consisting of the following chain of operations: median filtering (sigma = 5 pixels), background removal, H‐minima transformation, and contrast‐limited adaptive histogram equalization (CLAHE). More precisely, the median filter was used to smooth the images and reduce the noise, the background level was obtained with a histogram shape‐based method beyond the peak of the intensity distribution, H‐minima transform (H = 0.05) was additionally applied for further denoise, and CLAHE method was used to enhance the contrast of the grayscale image. Subsequently, processed images were binarized using Otsu thresholding method, which automatically identified the optimal intensity threshold to divide the images into foreground and background regions. This procedure enabled us to finally evaluate the fraction of area covered by the signals and the corresponding integrated density.

### 2.5 Tridimensional analysis of neuronal cytoskeleton within cortical organoids

Fluorescence 3D image stacks (133 × 133 × 8 µm^3^) of the cytoskeletal marker MAP2, acquired with a 100× objective, were segmented to perform morphological analysis of the cytoskeletal network using Huygens and MATLAB 2022b software. Before proceeding with binarization, we used Huygen Deconvolution Wizard to apply images deconvolution as a pre‐processing step to improve the contrast and resolution of the digital images recorded. The software automatically generated a theoretical P.S.F. computed from the microscope parameters. The resulting deconvolved 3D images were further processed and segmented with an automatic code developed in MATLAB, described in the following. Gaussian three‐dimensional filtering (sigma = 3 pixels), local background subtraction and Hessian‐based Frangi vesselness filter (r = 10 pixels) were applied to enhance tube‐like structures in the volumetric images data and extract the binary masks from the enhanced images via Otsu thresholding method. Finally, the resulting binary images were skeletonized via morphological thinning algorithms. We quantified the total number of separated fragments over the volume scanned and the average length of the fragments.

### 2.6 Immunostaining, image acquisition, and analysis of synapses on 2D cultures

Monolayer cortical cultures were fixed in 4% PFA solution for 15 min at room temperature and double‐washed in PBS (Thermo Fisher Scientific). Fixed cells were then permeabilized with PBS containing 0.2% Triton X‐100 (Sigma Aldrich) for 15 min and blocked with a solution containing 0.2% Triton X‐100 and 5% goat serum (Sigma Aldrich) for 20 min at room temperature. Cells were then incubated overnight at 4°C with primary antibodies reported in Table S1. The day after, cells were washed twice and incubated with secondary antibodies for 1 h at room temperature in the dark. AlexaFluor secondary antibodies (Thermo Fisher Scientific) were used at a concentration of 1:750 (Table S2), and Hoechst (Sigma Aldrich) was used for nuclei staining. Some samples were incubated with an antibody dilution buffer without primary antibodies to discriminate that the synaptic signals were not confounded with non‐specific binding of secondary antibodies in the sample. For synaptic markers quantification of 2D cortical networks, fluorescence images were acquired on an X‐Light V3 Spinning Disk Confocal module (Crest Optics) with a 100x/NA 1.45 oil objectives (Olympus) in stack with z‐step of 0.2 µm a Prime sCMOS camera (Photometrics), and a MetaMorph software (Molecular Devices). Signal deconvolution was applied through Huygens software (Huygens Professional, Scientific Volume Imaging). Quantification of synaptic markers has been carried out on Fiji software.^28^ Specifically, each signal has been adjusted by removing the background noise, and then a threshold has been performed to quantify both the area covered by each synaptic signal and the integrated density.

### 2.7 MitoTracker staining and confocal analysis

Mitochondria content and their morphology have been evaluated in live cells using MitoTracker Red FM (Invitrogen/Molecular Probes). 2D cultures have been obtained as previously reported. Briefly, up to five brain organoids have been dissociated at D70 and plated onto poly‐L‐ornithine/laminin‐coated dishes at a density of 80.000 cells per cm^2^. Cultures have been maintained in an appropriate neuronal culture medium (NMD‐A). On D100, cells were incubated with MitoTracker Red FM at a concentration of 300 nM for 30 min in a CO_2_ incubator at 37°C and double washed with NMD‐A medium, each for 5 min at 37°C, followed by two washes with PBS. Monolayer cortical cultures were fixed in 4% PFA solution for 15 min at room temperature and double‐washed in PBS. Fixed cells were then permeabilized with cold Methanol (Sigma Aldrich) for 7 min, then washed three times with PBS for 5 min each. An immunofluorescence protocol has been carried out as previously reported. MitoTracker signals were collected using an X‐Light V3 Spinning Disk Confocal module (Crest Optics) with a 100x/NA 1.45 oil objectives (Olympus) in a stack with a z‐step of 0.2 µm, a Prime sCMOS camera (Photometrics), and MetaMorph software (Molecular Devices). Signal deconvolution was applied through Huygens software (Huygens Professional, Scientific Volume Imaging). ImageJ software was utilized to analyze the images. Initially, Z‐projections were performed by merging approximately 10 planes, which improved the accuracy of the measurements. Following this, background removal was conducted, and brightness and contrast were adjusted to enhance the quality of the mitochondrial signal. Both the amount and lengths of individual mitochondria within axons were then measured using the “freehand” tool in ImageJ, specifically focusing on the axonal region by excluding mitochondria present in the soma. The lengths of individual mitochondria were averaged for each axon to obtain a representative value for further analysis.

### 2.8 Protein extraction and Western blot analysis

Cell lysates of D100 cortical organoids were homogenized with RIPA extraction buffer (50 mM Tris‐HCl pH 7.5, 150 mM NaCl, 1% NP40, 0.5% sodium dodecyl sulfate [SDS], 0.5% sodium deoxycholate, 1 mM ethylenediaminetetraacetic acid [EDTA], and 50 mM NaF) containing 1 mM phenylmethylsulfonyl fluoride (PMSF), 1× protease inhibitor cocktail (PIC), and 1 mM dithiothreitol (DTT) (all from Sigma‐Aldrich). After incubation on ice for 15 min, samples were centrifuged at 13,000 × *g* at 4°C for 30 min, and the supernatant containing total proteins was collected.

Protein concentrations were determined using the Bio‐Rad Protein Assay (Bio‐Rad Laboratories GmbH) following the manufacturer's instructions. Equal amounts of total protein (20 µg) were separated on 4%–12% NuPage Bis‐Tris gels (Life Technologies, Carlsbad, CA, USA), transferred to nitrocellulose membranes (Bio‐Rad, Hercules, CA, USA) for 1 h in Tris‐Glycine buffer, and blocked with 1× PBS–0.1% Tween20 solution containing 3% BSA (Serva). Membranes were then immunoblotted with specific antibodies (Table S3) and developed using enhanced chemiluminescent (ECL) blotting substrate (Advansta, San Jose, CA, USA). The quantification of protein bands was performed by densitometric analysis using the Image J software (1.47 version, NIH, Bethesda, MD, USA). Three different experiments (differentiation batches) were analyzed. Cortical organoids were pooled to provide population variability within each experiment.

### 2.9 PCR, RT‐PCR, and RT‐qPCR

Total RNA was extracted with the EZNA Total RNA Kit I (Omega Bio‐Tek) and retro‐transcribed using the iScript Reverse Transcription Supermix for reverse transcription quantitative polymerase chain reaction (RT‐qPCR; Bio‐Rad). Real‐time RT‐PCR was performed with iTaq Universal SYBR Green Supermix (Bio‐Rad) on a ViiA 7 Real‐Time PCR System (Applied Biosystems). A complete list of primers is provided in the Supplementary Material. (Table S4). ATP5O was selected as the housekeeping gene for normalization. To validate the suitability of ATP5O as a reference gene, we assessed its expression across eight independent batches of cortical organoids derived from both control and MAPT IVS10+16 mutant iPSC lines. RT‐qPCR was performed at five developmental time points (days 0, 30, 50, 70, and 100), and CT values were analyzed. Expression of ATP5O remained stable over time and showed no significant differences between genotypes, as determined by a two‐way analysis of variance (ANOVA) test (Figure S2). To further support its use as a stable reference, we also assessed the expression of ATP5O, RPLP0, and ACTB (β‐actin) at D0 and D100 in samples from four independent batches (two replicates each) derived from both genotypes. All three genes displayed consistent expression across time points and genotypes, as shown in Figure S2.

### 2.10 NanoString nCounter gene expression assays and analysis

nCounter gene expression assay (NanoString Technologies) was performed for the Human Neuropathology panel, including 760 genes covering pathways involved in neurodegeneration and other nervous system diseases, and 10 internal reference genes for data normalization. Briefly, panel codeset probes were hybridized with 100 ng of total RNA for 20 h at 65°C according to the manufacturer's instructions. Data were collected using the nCounter Digital Analyzer (NanoString). RNA counts were normalized using the geometric mean of the seven housekeeping genes (AARS, CCDC127, CNOT10, CSNK2A2, LARS, SUPT7L, TADA2B) that showed an average count > 450, after validation against positive and negative controls, using the nSolver 4.0 software (NanoString). The significant Differentially Expressed (DE) genes were selected by multiple *t*‐tests performed with Graph Pad Prism 6, with p‐value ≤ 0.05 and a fold change cutoff value of ≥ 1.5 or ≤ 0.66 for up‐modulated and down‐regulated genes, respectively. Gene ontology (GO) enrichment and pathway analysis were identified according to Database for Annotation, Visualization and Integrated Discovery (DAVID) functional annotation.^29^  The heat maps were performed for Hierarchical cluster using Average linkage and Pearson Distance Measurement Methods of Heatmapper tools (http://www.heatmapper.ca).

### 2.11 Calcium imaging recordings and data processing on whole cortical organoids

Calcium imaging acquisition on the whole cortical organoid was performed at room temperature using a custom fluorescence microscope. The high‐affinity calcium‐sensitive dye Fluo4‐AM (Invitrogen) was excited at a wavelength of 490 nm with the stable light source Lambda XL (Sutter Instrument) equipped with a Lambda 10‐B optical filter changer (Sutter Instrument). The emitted light was collected through a 525/50 nm filter. The fluorescence imaging was performed using a Zeiss Axio observer A1 inverted microscope (Zeiss) with a Zeiss A‐Plan 20x/NA 0.25 infinity corrected objective (Zeiss) and a CoolSNAPHQ2 camera (Photometrics). Images were acquired for 5 min at a sampling rate of 4 Hz and exposure time of 20 ms using Micromanager software. Neuronal cultures were incubated with the dye at a concentration of 5 µM for 30 min at 37°C in HEPES‐buffered external solution (NES) containing (in mM) 140 NaCl, 2.8 KCl, 2 CaCl_2_, 2 MgCl_2_, 10 HEPES, and 10 D‐glucose (pH 7.3 with NaOH; 290 mOsm). Calcium imaging data were processed using custom MATLAB codes.^23^, ^24^, ^30^ The first step involved identifying the regions of interest corresponding to neurons. An automated algorithm was developed to analyze the cumulative difference of the signal between frames of the time series in the frequency domain using 2D Fourier transform. The resulting matrix was filtered to remove high‐frequency noise, and the local maxima of the matrix were identified as neurons. Once the cell positions were determined, the fluorescence signals over time were collected. The calcium intensity traces were baseline corrected, normalized as ∆F/F_0_, and smoothed using a moving average filter. The processed traces were then analyzed to detect calcium transients and their characteristics. The criteria for automatic detection of a calcium transient were as follows: at the onset, both the fluorescence intensity and the slope of the trace showed an increase, and at the offset, the slope of the trace decreased, with a maximum time interval occurring between the onset and offset. The default threshold for peak amplitude was set to 2%. The algorithm provided the option to manually adjust any false or missing detections in the identified neuron positions and calcium transients on the filtered traces. Once the peaks were detected, an exponential fitting procedure was used to obtain amplitude, rising time, and decay time. The rising time estimation was particularly important for distinguishing fast calcium transients associated with neuronal activity from slower calcium signals originating from internal stores or other cell types. A threshold of τ* = 1.5 s was established to recognize and discard non‐neuronal signals. Amplitude, frequency, fraction of active neurons, and network synchrony (measured as the relative number of simultaneous events) were evaluated, exported to Microsoft Excel, and compared for different cell lines.

### 2.12 Statistical analysis

Statistical analysis, graphs, and plots were generated using GraphPad Prism 9 (GraphPad Software) and MATLAB 2016b (MathWorks). The Shapiro–Wilk normality test was conducted to assess whether the data sets followed a normal distribution. When the assumption of normality was not met, statistical significance was determined using the nonparametric two‐sided Mann–Whitney (MW) test. For comparisons involving more than two groups, the Kruskal–Wallis test was employed for nonparametric data, followed by Dunn's multiple comparison test where applicable (*p* = 0.05). In cases where data were normally distributed, a one‐way ANOVA with Sidak's multiple comparison test was used for multiple group comparisons, and a Student's *t*‐test (*p* = 0.05) was applied for two‐group comparisons unless stated otherwise. A two‐way ANOVA was conducted to assess the stability of gene expression across experiments, considering two factors: genotype and days of differentiation. Multiple comparisons were performed using Tukey's or Sidak's post hoc tests, as appropriate. Data are presented as mean ± standard error of the mean (SEM), with details on the number of cells, replicates, fields of view, cultures, and organoid batches provided in the corresponding figure legends. The sample size (*n*) was determined based on prior experimental data and statistical power analyses to ensure robust and reliable conclusions.

## 3 RESULTS

### 3.1 Tau isoforms dysregulation and neurites morphology impairment in MAPT‐mutant 3D cortical organoids

**FIGURE 1:**
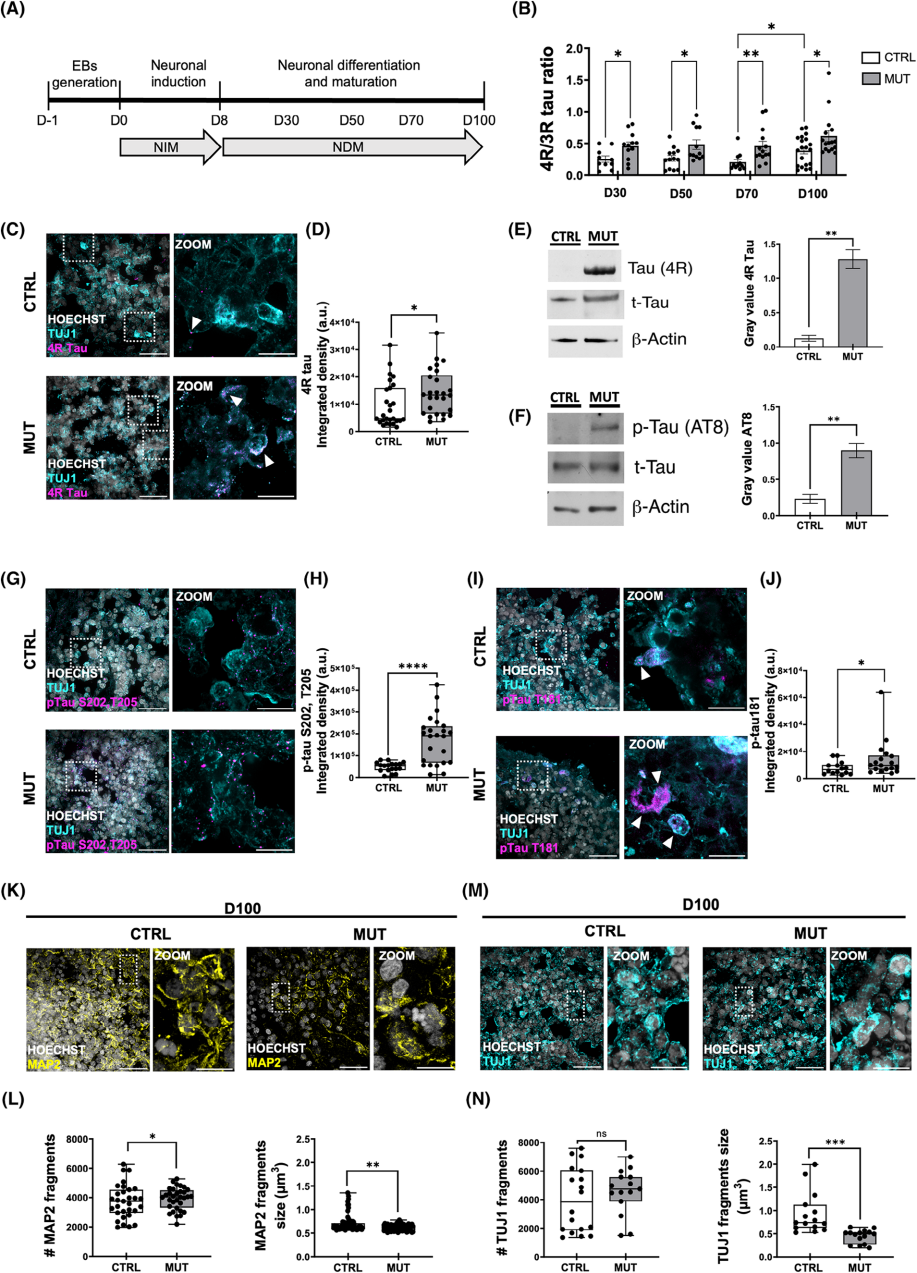
MAPT IVS10+16 mutation alters tau isoform balance and neurite morphology in cortical organoids. (A) Protocol timeline for the generation of cortical organoids, indicating the corresponding medium used at each developmental stage. (B) Scatter dot plot showing RT‐qPCR analysis of the 4R/3R tau ratio in control (CTRL, white bars) and tau‐mutant (MUT, gray bars) iPSC‐derived cortical organoids at different time points (D30, 50, 70, and 100; CTRL n = 18/8, MUT n = 16/7; replicates (10 organoids each)/batches, for each time point). Gene expression is normalized to the housekeeping gene ATP5O (**p < 0.001; * *p* < 0.05, two‐way ANOVA with Tukey test for multiple comparisons). (C) Representative fluorescence images of CTRL (top) and MUT (bottom) iPSC‐derived cortical organoids at D100, immunostained for the 4R tau isoform (magenta), neuronal beta3 tubulin (TUJ1, cyan), and Hoechst for nuclei visualization (gray). Scale bar, 30 µm. (D) Box plot representing the 4R tau signal as integrated density (ISD) within the field of view (FOV) in D100 CTRL and MUT cortical organoids (CTRL *n* = 27/3 FOVs/batches; MUT, *n* = 26/3 FOVs/batches; *p < 0.05, MW test). (E) Left, representative immunoblot of 4R tau isoform. Right, bar chart showing the amount of 4R protein in D100 CTRL and MUT cortical organoids. Actin was used as a protein loading control, and total tau was used to normalize the signal. Values are expressed as median ± SEM from four independent experiments (batches)(**p < 0.01, *t*‐test. (F) Left, representative immunoblot of phosphorylated tau at Ser202/Thr205 (AT8). Right, bar chart reporting the amount of AT8 protein in D100 CTRL and MUT cortical organoids. Actin was used as a protein loading control, and total tau was used for normalization. Values are expressed as median ± SEM from four independent experiments (batches)(** *p* < 0.01, *t*‐test). (G) Representative confocal images of CTRL (top) and MUT (bottom) cortical organoids at D100 immuno‐stained for anti‐AT8 (magenta), Tuj1 (cyan), and HOECHST (gray) for nuclei visualization. (H) Box plot representing the AT8 quantification as ISD within the FOV (CTRL *n* = 18/ 2 FOVs/batches; MUT *n* = 24/3 FOVs/batches; **** *p* < 0.0001 *t*‐test). (I) Representative confocal images of CTRL (top) and MUT (bottom) D100 organoids, immunolabeled for pTau181 (magenta), Tuj1 (cyan), and HOECHST (gray). Scale bar 30 µm. (J) Box plot representing the amount of pTau181 signal as integrated signal density within the FOV. (CTRL *n* = 15/2 FOVs/batches; MUT *n* = 18/3 FOVs/batches; *p < 0.05 MW test). (K) Representative confocal images of CTRL (left) and MUT (right) D100 organoids, immunolabeled for MAP2 (yellow) and HOECHST (gray). Scale bar 30 µm. (L) Box plots showing the quantification of the MAP2 signal as the number of fragments (left, CTRL *n* = 71/5; MUT *n* = 75/5; *p < 0.05 *t*‐test) and the fragment size/µm^3^ (right, CTRL *n* = 71/5; MUT *n* = 75/5; **p < 0.01 MW test). (M) Representative confocal images of CTRL (left) and MUT (right) D100 organoids immunolabeled for TUJ1 (cyan) and HOECHST (gray) (Scale bar 30 µm). (N) Box plots representing the quantification of the TUJ1 signal as the number of fragments (left) and the size of the fragments (left) per µm^2^ analyzed within FOV. (CTRL *n* = 15/2; MUT *n* = 15/3; NS *p* > 0.05 MW; ***p < 0.001 MW). ANOVA, analysis of variance; iPSC, induced pluripotent stem cell; MW, Mann–Whitney; RT‐qPCR, reverse transcription quantitative polymerase chain reaction.

We examined pathological tau species throughout the organoids’ growth phases to validate our 3D cortical organoid system as an effective and realistic in vitro microphysiological model for studying tauopathy. Our goal was to evaluate neuronal differentiation and maturation within the model and assess the influence of the familiar FTD‐linked IVS 10+16 MAPT mutation. Building upon the established Pasca's protocol,^25^ we promoted neuronal and glial differentiation and cortical organoid maturation by integrating heparin, ascorbic acid, BDNF, and GDNF to foster the proliferation and maturation of neuroepithelial‐like stem cells through the stimulation of collagen production^31^, ^32^ (Figure 1A and S1).

Initially, we investigated if the intronic IVS 10+16 MAPT mutation affected the balance between 3R and 4R tau isoforms during the maturation of cortical organoids. We performed Real‐Time PCR analysis of organoid development at different time points from D30 to D100 (Figure 1A). The gene expression analysis indicated that mutant organoids were characterized by a significantly higher 4R/3R ratio than controls from early stages (D30), due to exon 10 inclusion (Figure 1B). This difference remained significant over time, though it narrowed by D100 as control organoids increased 4R expression, reflecting normal developmental maturation. This imbalance was also observed at the protein level through immunofluorescence (Figure 1C, D) and Western blot (Figure 1E) analyses, revealing an increased expression of the 4R isoform in D100 tau‐mutant organoids. As it is known that the MAPT IVS 10+16 mutation, disrupting the equilibrium between 3R and 4R tau isoforms, leads to an increase in pathological tau variants,^6^, ^7^, ^33^ we further evaluated the presence of hyperphosphorylated tau in our in vitro disease model. This was accomplished using antibodies targeting phosphorylated tau at various serine and threonine sites. Western blot analysis revealed a significant increase in phosphorylated tau at Ser202/Thr205 (AT8) in tau‐mutant organoids (D100) compared to controls (Figure 1F). Immunofluorescence analysis confirmed the increase in AT8 staining (Figure 1G, H) and also revealed the increase of phosphorylated tau at Thr181 (pTau181) (Figure 1I, J).

Considering tau's critical role in microtubule stabilization and how its hyperphosphorylation undermines this function,^34^, ^35^, ^36^, ^37^ we also assessed neurite morphology by examining neuronal cytoskeletal proteins. Immunostaining for MAP2 (Figure 1K, L) and β3‐tubulin (TuJ1; Figure 1 M, N) demonstrated that neurites in tau‐mutant organoids exhibited a fragmented morphology, with smaller neurite fragments compared to isogenic controls. This suggests that the FTD‐associated tau mutation compromises neuronal integrity and/or neurite elongation during early developmental stages. Although the degree of MAP2 fragmentation is relatively modest, it is statistically significant and aligns with a broader pattern of neuronal structural dysfunction. These findings indicate that the intronic MAPT IVS 10+16 mutation triggers imbalances in tau isoform levels and elevated tau phosphorylation from the early stages of development, potentially affecting the neuronal cytoskeleton in 3D cortical organoids.

### 3.2 Intronic MAPT IVS 10+16 mutation impairs cortical organoid development and maturation

The observation that this intronic tau mutation impacts neurites morphology at D100 of cortical organoid maturation, combined with tau's established role in shaping different aspects of neuronal development, such as axonal guidance, synaptogenesis, and neuronal migration,^34^ led us to investigate the mutation's effect on key genes at two critical organoids developmental points (D50 and D100). By applying nCounter Nanostring technology for total RNA examination in both the control and tau‐mutants organoids using the Neuropathology Panel (which includes 760 genes), we detected significant gene expression alterations: 130 genes at D50 and 243 genes at D100 were DE due to the tau mutation (*p* < 0.05) (see Figure S3A, B, and Tables S5 and S6).

Initially, we analyzed gene expression modulation throughout the development of organoids obtained from both iPSC lines. In the control organoids, we noted a differential expression of 183 genes from D50 to D100 (Figure S4A and Table S7).

GO term enrichment analysis of the 164 upregulated genes at D100 with respect to D50 showed categories chiefly associated with synaptic transmission (biological processes, GO: BP), protein binding (molecular function, GO: MF), and neuronal shape development (cellular component, GO: CC). Conversely, there was a reduction in 19 genes, predominantly related to the cell cycle (Figure S4B), indicating the time course of cortical organoid maturation.

Within the tau‐mutant organoids, we identified alterations in the expression levels of 180 genes at D100 compared to D50, with 114 genes upregulated and 66 genes downregulated (see Figure S5A, Table S8). These changes were only partially related to neuronal maturation and differentiation. Among the upregulated genes, categories associated with synaptic transmission were observed (Figure S5B); however, these categories were also present among the downregulated genes (Figure S5B). Furthermore, there was no downregulation of genes related to the cell cycle, indicating a persistent proliferation state in mutant organoids at D100.

**FIGURE 2:**
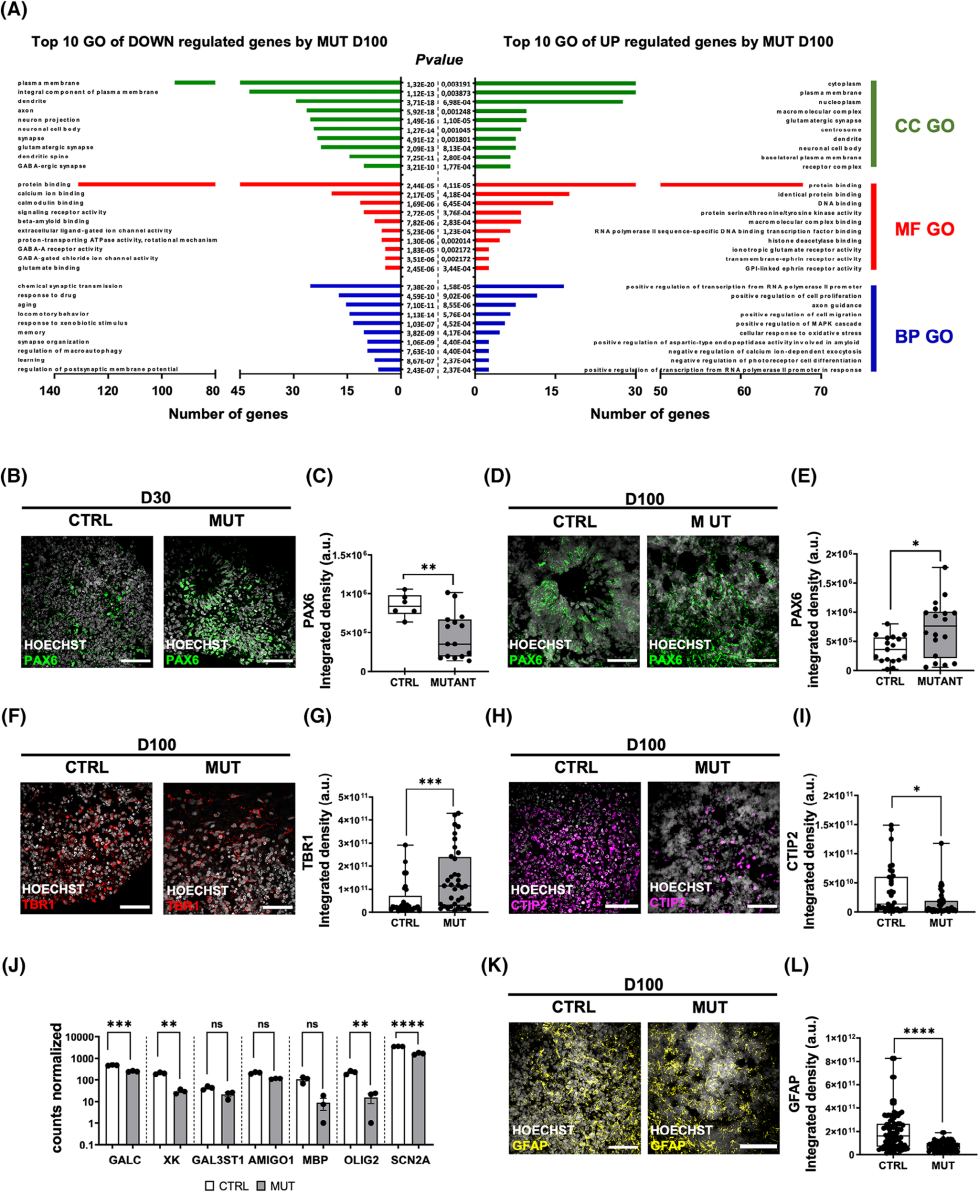
Intronic tau mutation delays cortical maturation. (A) GO enrichment analysis of down‐ (left) and up‐ (right) regulated genes by D100 MUT respect to CTRL organoids analyzed by DAVID database. An overview of the top 10 significantly enriched terms in three categories: cellular component (CC), molecular function (MF), and biological process (BP). The number of genes involved in a GO process is shown in the x‐axis. The cutoff of the p‐value was set to 0.05. (B) Representative confocal images of CTRL (left) and MUT (right) cortical organoids at D30 immunolabeled for PAX6 (green) and HOECHST (gray) for nuclei visualization. Scale bar, 30 µm. (C) Box plot representing the amount of PAX6 as ISD within each FOV (CTRL *n* = 20/2 FOVs/batches, white; MUT *n* = 17/2 FOVs/batches, gray; *t*‐test **p < 0.01). (D) Representative confocal images of CTRL (left) and MUT (right) cortical organoids at D100 immunolabeled for neuronal progenitor marker PAX6 (green) and HOECHST (gray). Scale bar, 30 µm. E) Box plot representing the amount of PAX6 as ISD within each FOV (CTRL *n* = 30/3 FOVs/batches; MUT *n* = 29 /3 FOVs/batches; MW test p* < 0.05). (F) Representative confocal images of D100 CTRL (left) and MUT (right) cortical organoids immunolabeled for TBR1 (red) and HOECHST (gray) for nuclei visualization. Scale bar, 30 µm. (G) Box plot reporting the amount of TBR1 as ISD within each FOV (CTRL *n* = 29/2 FOVs/batches; MUT *n* = 33/3 FOVs/batches; MW test *** *p* < 0.001). (H) Representative confocal images of CTRL (left) and MUT (right) D100 organoids immunolabeled for CTIP2 (magenta) and HOECHST (gray). Scale bar, 30 µm. (I) Box plot representing the quantification of CTIP2 signal as ISD within each FOV (CTRL *n* = 37/2 FOVs/batches; MUT *n* = 38 /3 FOVs/batches; MW test p* < 0.05). (J) Scatter dot plot showing the expression level of genes related to the myelination process in D100 CTRL and MUT organoids (CTRL *n* = 3/1 batches; MUT n = 3/1 FOVs/ batches; ***p < 0.0001, *** *p* < 0.001, **p < 0.01, *p < 0.05 One way ANOVA test plus Sidak for multiple comparisons). (K) Representative confocal images of CTRL (left) and MUT (right) D100 organoids immunolabeled for GFAP (yellow) and HOECHST (gray). Scale bar, 30 µm. (L) Box plot representing the quantification GFAP signal as ISD within each FOV (CTRL *n* = 93/3 FOVs/batches; MUT *n* = 62/3 FOVs/batches; MW test p**** < 0.0001). ANOVA, analysis of variance; CTRL, control; DAVID, Database for Annotation, Visualization and Integrated Discovery; FOV, field of view; GFAP, glial fibrillary acidic protein; GO, gene ontology; ISD, integrated signal density; MUT, mutant; MW, Mann–Whitney.

We then analyzed the genes DE at D100 between control and tau‐mutant organoids (tot 243 genes) (Figure S3B and Table S6). GO term enrichment analysis of the down‐regulated subset at D100 in tau‐mutant organoids with respect to their isogenic controls (163 genes) revealed categories mainly related to excitatory and inhibitory synapses, neuronal projection, dendrites, and axon (GO: CC); calcium homeostasis, glutamate and gamma‐aminobutyric acid (GABA) homeostasis and signaling (GO: MF); nervous system development (GO: BP) (Figure 2A, left). When looking at the 80 upregulated genes in tau‐mutant organoids, the GO term enrichment analysis showed categories mainly related to the centrosome, basolateral plasma membrane, and cytoplasm (GO: CC); protein, and nucleic acid binding (GO: MF); positive regulation of cell proliferation and intracellular cascades linked to cellular stress (GO: BP) (Figure 2A, right). These data suggest a delay in tau‐mutant cortical organoids maturation and indicate that tau targets include genes that play crucial roles in neuronal and neuronal network development and maturation.

At the protein level, immunofluorescence analysis of the neural progenitor marker PAX6 showed lower levels of PAX6‐positive cells in tau‐mutant cortical organoids at D30 (Figure 2B, C), while at D100 (Figure 2D, E) tau‐mutant organoids were characterized by higher PAX6 expression compared to their isogenic control, confirming a delay in the maturation process and a delayed persistent presence of proliferative progenitors during organoids maturation, as also supported by the higher expression levels of cell cycle‐related genes PCNA and CCNA2 (Figure S6A). To investigate neuronal fate commitment, we analyzed the expression of cortical neuronal markers TBR1 and CTIP2. At D100, tau‐mutant organoids exhibited an increased presence of immature post‐mitotic neurons expressing TBR1 (Figure 2F, G) and a reduced number of layer 6 cortical neurons marked by CTIP2 (Figure 2H, I) compared to controls. These findings suggest that the intronic MAPT IVS 10+16 mutation delays neuronal subtype specification, further disrupting cortical development. Furthermore, we also observed that the glial development was delayed in tau‐mutant organoids, with downregulation of genes related to the myelination process (GALC, XK, GAL3ST1, AMIGO1, MBP, OLIG2, SCN2A9) (Figure 2J), and reduced GFAP protein expression at D100 (Figure 2K, L). These data indicate that the tau IVS 10+16 mutation strongly delays cortical development, affecting both the neuronal and the glial maturation.

### 3.3 Synaptic and network functionality deficits in Tau‐mutant cortical organoids

**FIGURE 3:**
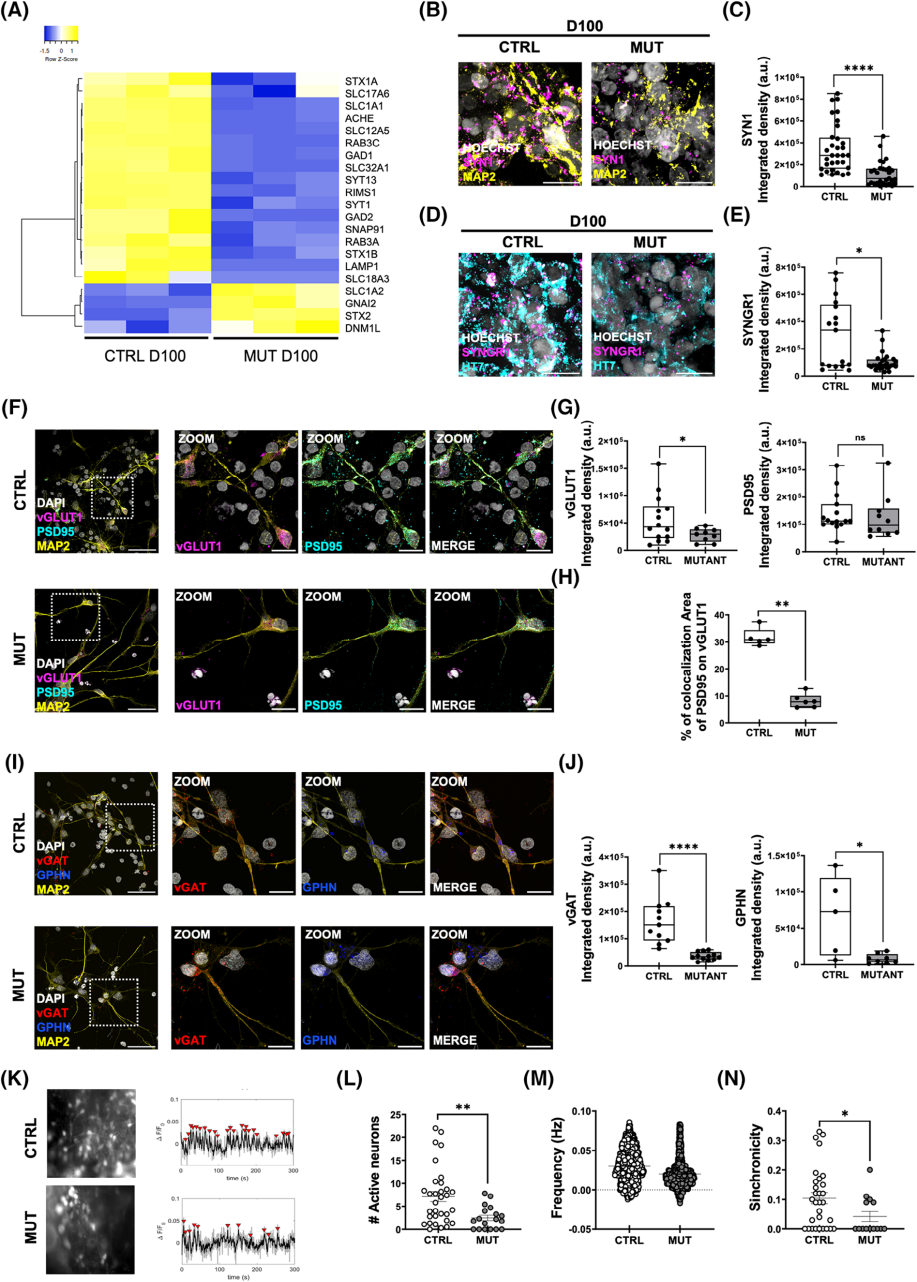
IVS10+16 tau mutation impairs synaptic maturation. (A) Heat map of unsupervised hierarchical clustering of 21 «Synaptic Pathways» genes between D100 MUT and CTRL organoids. Average linkage and Pearson Distance Measurement Methods were performed for Hierarchical cluster using Heatmapper tools (http://www.heatmapper.ca). The significant genes are selected by multiple *t*‐tests performed with Graph Pad Prism 6, with *p*‐value ≤ 0.05. (B) Representative confocal images of D100 CTRL (left) and MUT (right) organoids immunolabeled for synapsin1 (SYN1) (magenta), MAP2 (yellow), and HOECHST (gray). Scale bar 10 µm. (C) Box plot representing SYN1 signal as ISD within each FOV (CTRL *n* = 32/3 FOVs/batches; tau‐MUT *n* = 30/4 FOVs/batches; MW test ****p < 0.0001). (D) Representative confocal images of CTRL (left) and MUT (right) D100 organoids immunolabeled for Synaptogyrin‐1 (SYNGR1) (magenta), HT7 (cyan) and HOECHST (gray). Scale bar 10 µm. (E) Box plot representing the amount of SYNGR1 as ISD for each FOV (CTRL *n* = 17/3 FOVs/batches; MUT *n* = 20/4 FOVs/batches; *p < 0.05; MW test). (F) Representative images of CTRL (top) and MUT (bottom) 2D cortical neurons immunolabeled for VGLUT1 (magenta), MAP2 (yellow), PSD95 (cyan), and HOECHST (gray) at D100. Scale bar 30 µm, 10 µm. (G) Box plots representing the amount of glutamatergic synaptic proteins as ISD for each FOV: VGLUT1 (left, CTRL *n* = 14/3 FOVs/batches; MUT *n* = 9/1 FOVs/batches; *p < 0.05 *t*‐test); PSD95 (right, CTRL *n* = 13/3 FOVs/batches; MUT *n* = 10/1 FOVs/batches; NS MW test). (H) Box plot representing VGluT1 and PSD95 colocalization signal within the FOV (CTRL *n* = 5/2 FOVs/batches; MUT *n* = 6/2 FOVs/batches; **p < 0.01 MW test). (I) Representative images of CTRL (top) and MUT (bottom) 2D cortical neurons immunolabeled for VGAT (red), MAP2 (yellow), GPHN (blue), and HOECHST (gray) at D100. Scale bar 30 µm, 10 µm. (J) Box plots representing the amount of GABAergic synaptic proteins as ISD for each FOV: VGAT (left, CTRL *n* = 11/2 FOVs/batches; MUT *n* = 12/2 FOVs/batches; ****p < 0.0001 *t*‐test), GPHN (right, CTRL *n* = 8/2 FOVs/batches; MUT *n* = 8/1 FOVs/batches; *p < 0.05 *t*‐test). (K) Representative traces of spontaneous calcium oscillations recorded CTRL (top) and MUT (bottom) cortical organoids at D100 of the differentiation protocol. Dot plots representing (L) the number of active neurons, (M) the frequency, and (N) the synchronicity of spontaneous calcium oscillation in CTRL (empty circles) and MUT (filled circles) cortical organoids (CTRL 47/organoids/batches; MUT 29/organoids/batches; *p < 0.05, **p < 0.01, MW test). CTRL, control; FOV, field of view; ISD, integrated signal density; MUT, mutant; MW, Mann–Whitney.

Among the genes DE in tau‐mutant organoids at D100, we observed a significant downregulation of transcripts associated with glutamatergic and GABAergic synapses and those linked to synaptic structure (Figure 3A). Immunofluorescence analysis of the pan‐synaptic markers synapsin‐1 (SYN1, Figure 3B, C) and synaptogyrin‐1 (SYNGR1, Figure 3D, E) revealed reduced expression levels of both proteins in tau‐mutant cortical organoids, corroborating the Nanostring data that suggest impaired synapses formation. Notably, normalized MAPT transcript counts show that while MAPT expression levels were comparable between control and mutant organoids at D50, by D100, MAPT expression significantly increased in controls, consistent with ongoing neuronal maturation, whereas it decreased in mutants, suggesting impaired maturation (Figure S6B).

To deeply investigate the excitatory and inhibitory synapses, immunostaining analysis was performed in cortical neurons obtained after dissociating cortical organoids at D70 and cultured for 30 days (see the scheme in Figure S1A). Labeling for glutamatergic and GABAergic synaptic markers revealed that the tau mutation reduced the formation of both synapses. Specifically, for glutamatergic synapses, we observed lower levels of the presynaptic marker (VGluT1, Figure 3F, G), and reduced colocalization (Figure 3H) with the post‐synaptic (PSD95, Figure 3F, G) protein. When we looked at GABAergic synapses, we observed lower levels of pre‐ (VGAT, Figure 3I, J) and post‐ (Gephyrin, Figure 3I, J) synaptic proteins, suggesting an impaired neuronal network development in tau‐mutant organoids.

To test the network functionality, we recorded spontaneous calcium transients in whole control and tau‐mutant cortical organoids at D100, loading organoids with Fluo‐4 M, and acquiring large fields of view (260 × 260 microns) at a high sample rate (4 Hz). Data from 5 min time‐lapse recordings (Supplementary Videos 1, 2) were collected and analyzed using a custom‐made algorithm to identify cells, select active zones, and extract functional properties such as amplitude, frequency, kinetic parameters, and network synchronicity.^23^ For each field of view, we divided active cells into two populations using the 1.5 s value as the rising time threshold of spontaneous calcium transients, and we analyzed the properties of the spontaneous activity of fast cells (rise time < 1.5 s), most probably neurons (Figure 3K). As represented in the dot plots, in tau‐mutant organoids, we found a smaller number of active neurons (Figure 3L), characterized by weaker spontaneous activity. Indeed, we found that both the frequency (Figure 3 M) and the synchronicity (Figure 3N) of calcium events were significantly lower in tau‐mutant organoids with respect to isogenic control. The analysis of the peaks showed that peak amplitude and kinetic parameters were similar in tau‐mutant and control organoids (Figure S7A‐C).

To complement these findings, we also performed calcium imaging on 2D neuronal cultures derived from dissociated cortical organoids. These cultures contain neurons originating from all organoid layers, thus providing a more comprehensive view of activity across the entire structure. As shown in Figure S7, D100 mutant neurons exhibited spontaneous calcium transients with significantly reduced amplitude (Figure S7D) and frequency (Figure S7E) compared to isogenic controls.

Altogether, these data suggest that intronic tau mutation leads to significant impairments in synaptic development and network functionality, highlighting the critical role of tau in maintaining synaptic integrity and neuronal connectivity in cortical organoids.

### 3.4 Molecular pathways and mitochondrial dysfunction in Tau‐mutant cortical organoids

To dissect the possible molecular pathways leading to the observed impairment in cortical development, we matched the differentially regulated genes in tau‐mutant organoids (at D100) with the targets of tau described by interactome analysis.^10^

**FIGURE 4:**
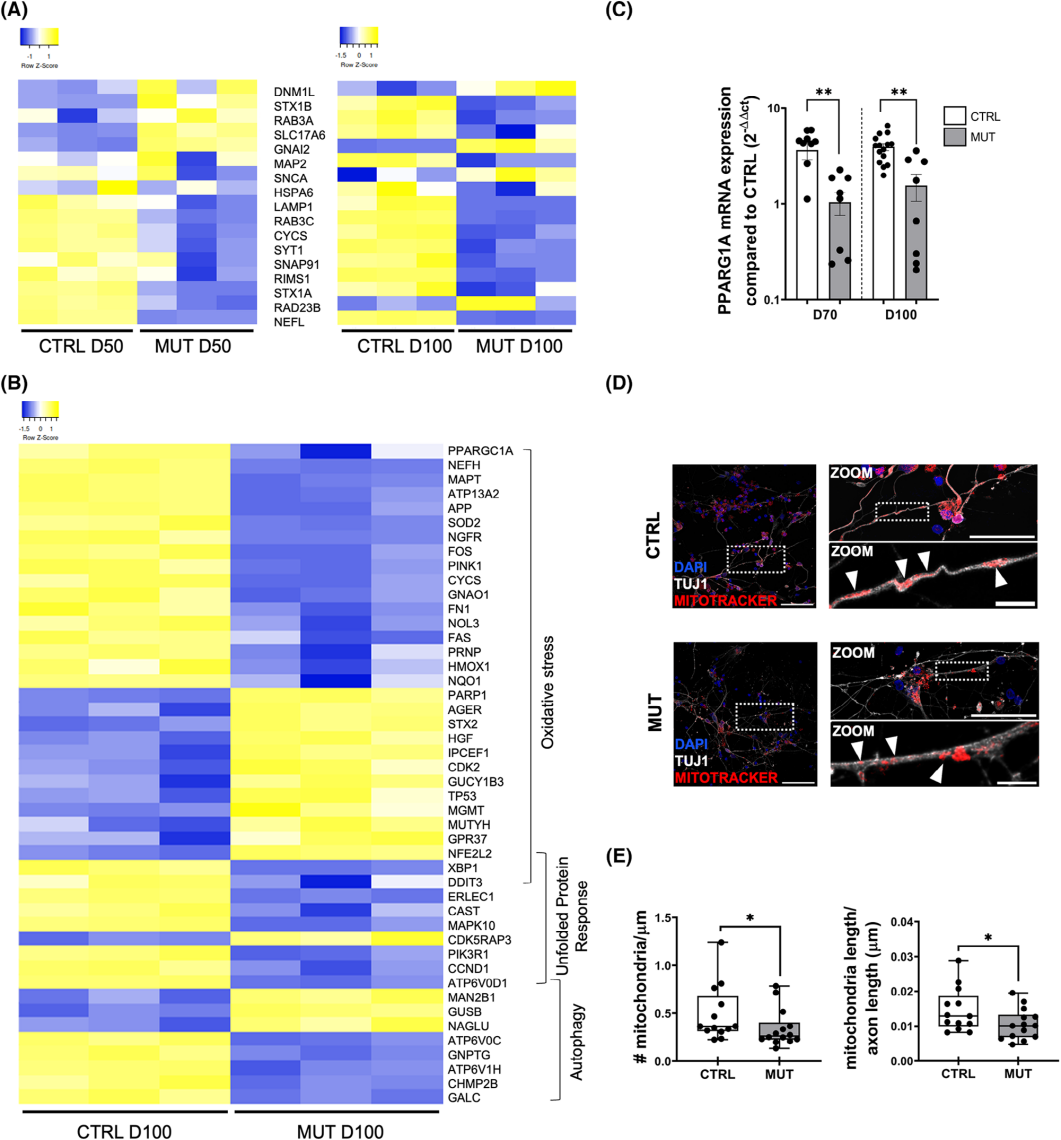
MAPT IVS 10+16 mutation induces mitochondrial alterations. (A) Heat map of supervised hierarchical clustering of 17 «TAU signature» genes between D50 CTRL vs D50 MUT and D100 CTRL vs D100 MUT. Average linkage and Pearson Distance Measurement Methods were performed for the Hierarchical cluster using Heatmapper tools (http://www.heatmapper.ca). The significant genes are selected by multiple *t*‐test performed with Graph Pad Prism 6, with *p* value ≤ 0.05. (B) Heat map of supervised hierarchical clustering of 46 «Oxidative Stress», «Unfolded Protein Response» and «Autophagy» genes between D100 CTRL vs D100 MUT. Average linkage and Pearson Distance Measurement Methods were performed for the Hierarchical cluster using Heatmapper tools (http://www.heatmapper.ca). The significant genes are selected by multiple *t*‐tests performed with Graph Pad Prism 6, with *p*‐value ≤ 0.05 and a fold change cutoff value of ≥1.5 or ≤0.66 for up‐modulated and downregulated genes, respectively. (C) Scatter dot plot showing the quantitative real‐time PCR analysis of PPARGC1A in CTRL and MUT organoids at D70 (CTRL *n* = 10/6, MUT *n* = 9/4; replicates (10 organoids each)/batches for each time point) and D100 (CTRL *n* = 15/6, MUT *n* = 8/3; replicates (10 organoids each)/batches for each time point). Gene expression is normalized to the housekeeping gene ATP5O (** *p* < 0.001, one‐way ANOVA plus Sidak for multiple comparisons). (D) Representative images of CTRL (top) and MUT (bottom) 2D cortical neurons immunolabeled with MitoTracker Red FM (red) and TUJ1 for neuronal cytoskeleton (gray), DAPI (blue) for nuclei visualization at D100. Scale bar: 50 µm; 30 µm, and 10 µm. (E) Box plots representing the number of mitochondria (left) and mitochondria length (right) in same‐length segments of TUJ1‐positive branches (CTRL *n* = 12/2 neurites/batches; MUT *n* = 13/2 neurites/batches; *p < 0.05 MW test). ANOVA, analysis of variance; CTRL, control; MUT, mutant; MW, Mann–Whitney; PCR, polymerase chain reaction.

We identified 17 “tau signature” genes differentially regulated in tau‐mutant organoids at both developmental time points D50 and D100 (Figure 4A). These genes are involved in synaptic vesicle‐associated functions, cytoskeletal structure, and mitochondrial and lysosome functions, thus confirming the impairment of neuronal network development and suggesting a possible defect of mitochondrial functions in tau‐mutant cortical organoids.

Indeed, out of the 243 genes DE at D100 between control and mutant organoids, 46 were associated with autophagy, unfolded protein response, and oxidative stress, all linked to mitochondrial function (Figure 4B).

Our attention was particularly drawn to the significant downregulation of the mitochondrial biogenesis master gene, PPARGC1A, observed at D100 in mutant organoids (*p*‐value = 0.021) (Figure 4B and Table S6. Although no significant difference in PPARGC1A expression was noted at D50 (Table S5), closer examination using Real‐Time PCR at the intermediate time point of D70 and final time point of D100 revealed a marked down‐regulation of the PPARGC1A transcript in tau‐mutant organoids (Figure 4C). This finding supports the Nanostring data at D100 and underscores the period between D50 and D70 as potentially critical for cortical development.

We thus employed MitoTracker labeling to evaluate mitochondrial quantity and structure within 2D cortical cultures at D100. After mitochondrial labeling, the cells underwent fixation and were stained with the neuronal TuJ1 antibody (Figure 4D). The analysis of mitochondria in same‐length segments of TuJ1‐positive branches showed that in tau‐mutant neurons, neurites contained fewer and smaller mitochondria (Figure 4E) compared to those in isogenic control neurons, indicating that the intronic MAPT IVS 10+16 mutation impairs mitochondrial biogenesis and maturation in cortical neurons. Although small, these differences in mitochondrial number and size are statistically significant and align with the reduction of mitochondrial genes.

### 3.5 Bezafibrate treatment mitigates mitochondrial dysfunction and enhances neuronal integrity in Tau‐mutant cortical organoids

We thus explored the efficacy of pharmacological activation of mitochondrial biogenesis in tau‐mutant cortical organoids, characterized by low expression of PPARGC1A, the gene encoding the master regulator protein PGC1α. For this purpose, we employed BZ, a peroxisome proliferator‐activated receptor (PPAR) agonist. Indeed, BZ is proposed as a therapeutic option for neurological and mitochondrial disorders and has shown promise in mitigating tau pathology.^38^, ^39^

Since we observed a timely decrease in PPARGC1A expression during the critical maturation phase (D50‐D70) in tau‐mutant organoids, we administered 5 µM BZ within this specific timeframe (Figure 1). The impact of BZ treatment was then assessed at D100, with comparative analyses conducted on treated and untreated tau‐mutant cortical organoids, alongside their isogenic controls, derived from parallel experimental batches.

**FIGURE 5:**
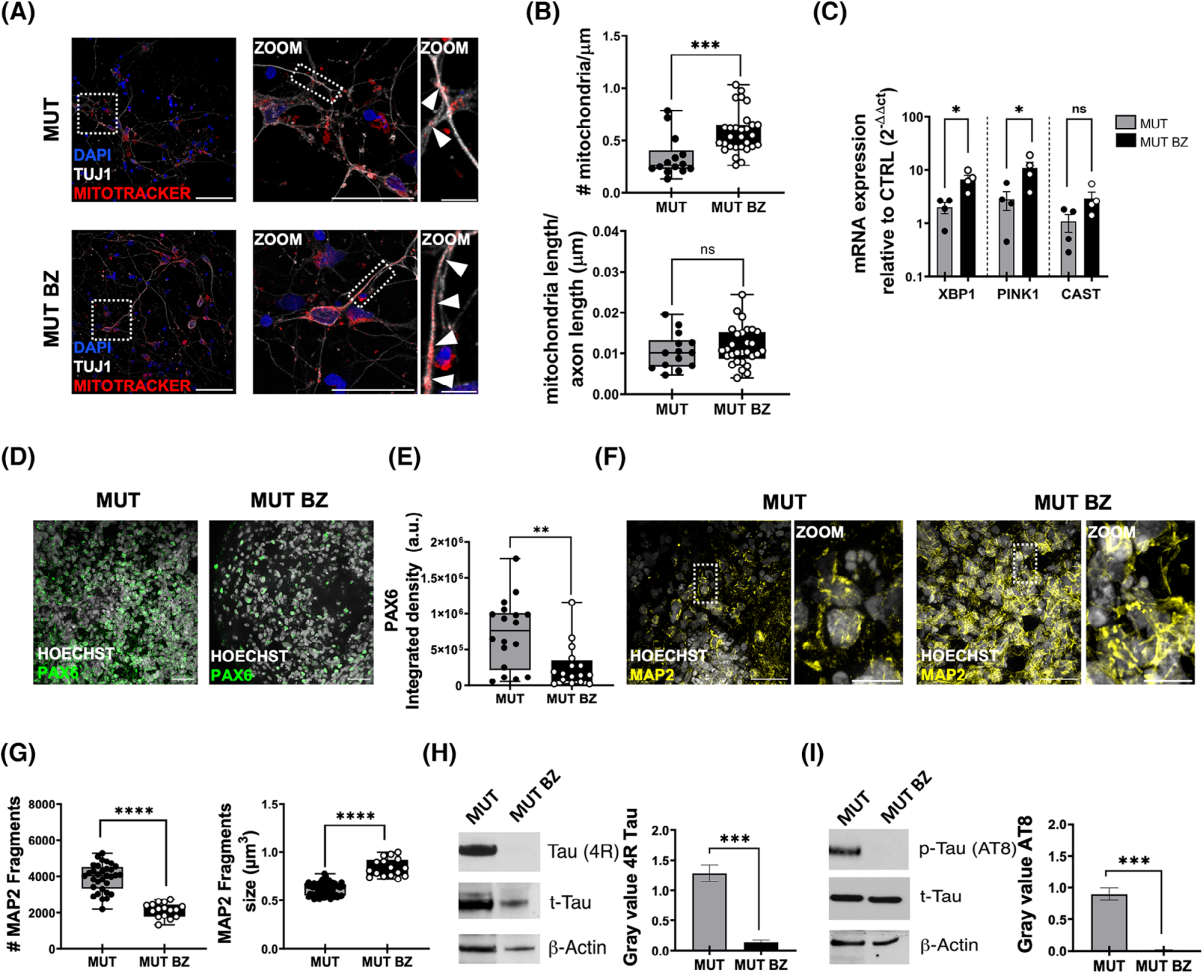
Bezafibrate (BZ) treatment partially reverses the effects of MAPT IVS 10+16 on mitochondria and neuronal cytoskeleton. (A) Representative images of untreated (top) and BZ treated (bottom) MUT 2D cortical neurons immunolabeled with MitoTracker Red FM (red) and TUJ1 (gray) at D100, DAPI (blue). Scale bar 30 µm; 5 µm. (B) Box plots representing the number (top) and the length (bottom) of mitochondria in same‐length segments of TUJ1‐positive branches (MUT *n* = 13/2 neurites/batches (gray bars); MUT BZ *n* = 29/3 neurites/batches (black bars); ***p < 0.001, NS *p* > 0.05 MW test). (C) Scatter dot plot showing the quantitative real‐time PCR analysis of XBP1, PINK1, and CAST gene in untreated and treated (BZ) MUT D100 organoids (MUT *n* = 4/2, MUT BZ *n* = 4/2; replicates [10 organoids each]). Gene expression is normalized to the housekeeping gene ATP5O (*p < 0.05, NS *p* > 0.05 *t*‐test). (D) Representative images of MUT (left) and MUT BZ (right) D100 organoids immunolabeled with PAX6 (green) and HOECHST (gray). Scale bar 30 µm. (E) Box plot showing the amount of PAX6 as ISD for each FOV (MUT *n* = 29/3 FOVs/batches; MUT BZ *n* = 16/3; p** < 0.01 MW test). (F) Representative images of MUT (left) and MUT BZ (right) D100 organoids immunolabeled with MAP2 (yellow) and HOECHST (gray). Scale bar: 30 µm, 10 µm. (G) Box plot showing the quantification of the MAP2 signal as the number of fragments (left) and fragment size/µm^3^ (right) (MUT *n* = 75/5; MUT BZ *n* = 16/2; **** *p* < 0.0001 MW test). (H) Left, representative immunoblot of 4R tau isoform. Right, bar graph showing the amount of 4R protein in D100 untreated and BZ treated MUT organoids. Actin was used as a protein loading control, and total tau was used to normalize the signal. Values are expressed as median ± SEM from three independent experiments (batches); *** *p* < 0.001 MW test. (I) Left, representative immunoblot of phosphorylated tau at Ser202/Thr205 (AT8). Right, bar graph reporting the amount of AT8 protein in D100 untreated and BZ treated MUT organoids. Actin was used as a protein loading control, and total tau was used for normalization. Values are expressed as median ± SEM from three independent experiments (batches); *** *p* < 0.001, MW test. FOV, field of view; ISD, integrated signal density; MUT, mutant; MW, Mann–Whitney; PCR, polymerase chain reaction.

First, we found that BZ treatment of tau‐mutant cortical organoids effectively normalized mitochondrial parameters. Specifically, Mitotracker labeling (Figure 5A) demonstrated that BZ application increased both the quantity and the dimension of neuritic mitochondria (Figure 5B) in tau‐mutant cortical neurons, with values similar to those observed in control cultures (Figure 4E). Furthermore, real‐time PCR analysis showed that the expression levels of mitochondrial genes downregulated in tau‐mutant organoids—XBP1 and PINK1 (see Figure 4B), associated with oxidative stress, the unfolded protein response, and autophagy—were significantly increased following BZ treatment (Figure 5C). CAST expression showed an upward trend, though this did not reach statistical significance. In contrast, PGC1α levels remained unchanged (*n* = 8 replicates/4 batches, *p* = 0.94).

We also found that BZ treatment of tau‐mutant organoids reduced PAX6 expression to the level of control organoids (Figure 5D, E), supporting the idea that the rescue of mitochondrial biogenesis favors neuronal differentiation and maturation. In line with these results, immunostaining for the neurite marker MAP2 demonstrated that BZ treatment rescued the neurite phenotype, decreasing the number of MAP2 fragments (Figure 5F, G) and augmenting their length (Figure 5F, G). Moreover, although BZ did not alter the 4R/3R ratio at the transcript level (Figure S8A), it restored 4R tau protein expression (Figure 5H, and Figure S8B) and significantly reduced phosphorylated tau at Ser202/Thr205 (AT8) (Figure 5I) to levels comparable with controls. These effects suggest a beneficial impact on neuronal maturation and neurite complexity.

Altogether, these findings suggest that impaired mitochondrial biogenesis contributes to reduced neuronal differentiation and maturation, and that its restoration supports neuronal development and a physiological tau expression profile. While bezafibrate did not directly correct the splicing defect, it enhanced mitochondrial function and proteostasis, which in turn partially normalized 4R tau expression, reduced tau phosphorylation, and promoted neuronal maturation and function.

### 3.6 Bezafibrate treatment rescues synaptic maturation and neuronal functions

**FIGURE 6:**
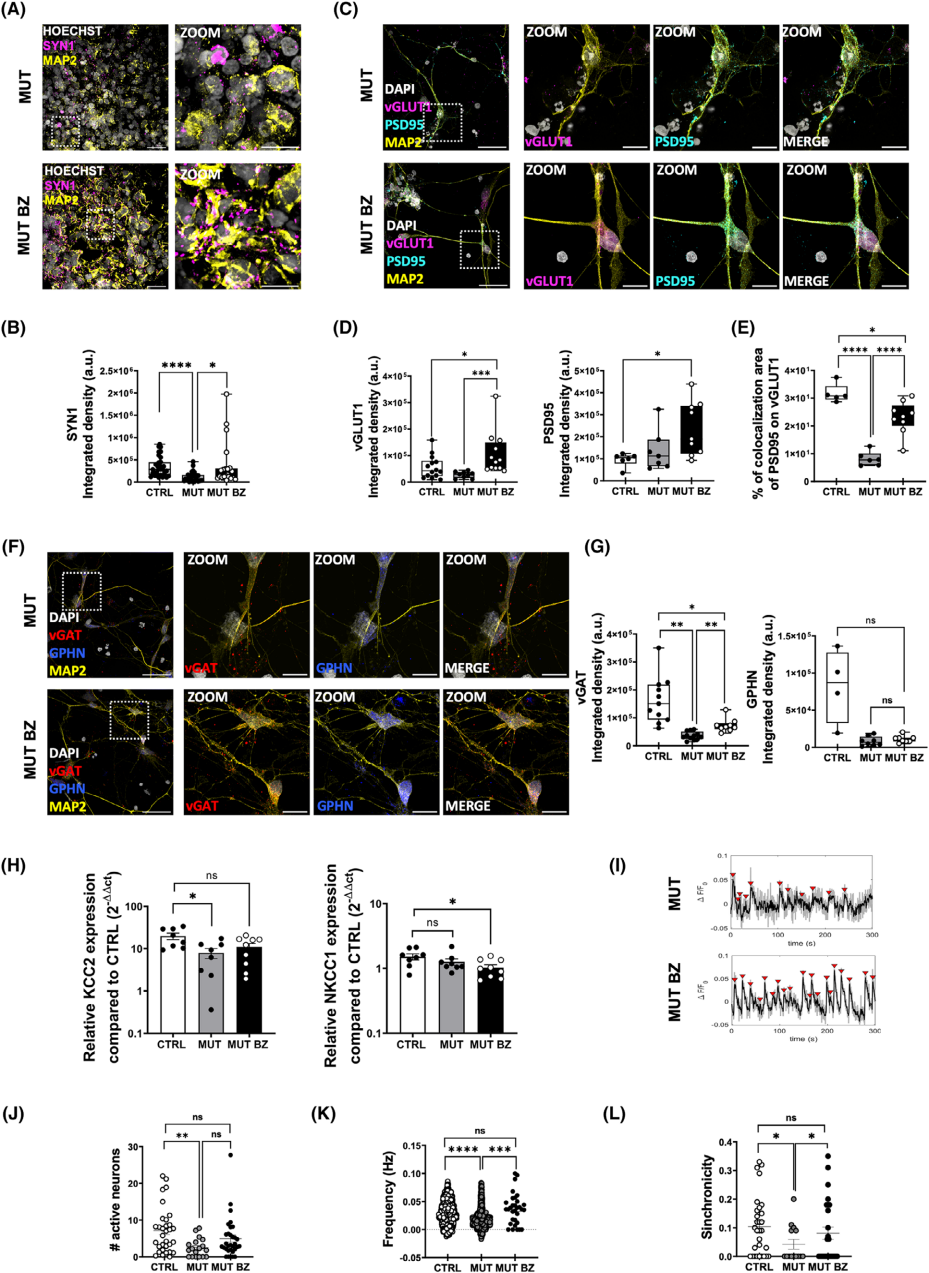
Bezafibrate treatment rescues synaptic functionality. (A) Representative images of MUT (top) and MUT BZ (bottom) D100 organoids immunolabeled for SYN1 (magenta), MAP2 for neuronal cytoskeleton (yellow), and HOECHST (gray). Scale bar: 30 and 10 µm. (B) Box plot representing the amount SYN1 as ISD for each FOV (CTRL *n* = 32/3 FOVs/batches; MUT *n* = 30/4 FOVs/batches; MUT BZ *n* = 21/2 FOVs/batches; ****p < 0.0001, *p < 0.05, Kruskal–Wallis with Dunn's comparison). (C) Representative images of MUT (top) and MUT BZ (bottom) 2D cortical neurons at D100 immunolabeled for VGLUT1 (magenta), MAP2 (yellow), PSD95 (cyan), and HOECHST (gray). Scale bar 30 µm, 10 µm. (D) Box plots representing the amount of glutamatergic synaptic proteins as ISD for each FOV: VGLUT1 (left, MUT *n* = 7/2; MUT BZ, *n* = 12/2 FOVs/batches; *p < 0.05, **p < 0.0001, Kruskal–Wallis test with Dunn's comparison), PSD 95 (right, MUT *n* = 10/2; MUT BZ *n* = 14/2 FOVs/batches; *p < 0.05; Kruskal Wallis test with Dunn's comparison). (E) Box plot representing VGLUT1 and PSD95 colocalization (CTRL *n* = 5/2 FOVs/batches; MUT *n* = 6/2 FOVs/batches; MUT BZ *n* = 9/3 FOVs/batches; *p < 0.05; ****p < 0.0001 one‐way ANOVA plus Sidak for multiple comparisons). (F) Representative images of MUT (top) and MUT BZ (bottom) 2D cortical neurons immunolabeled for VGAT (red), MAP2 (yellow), GPHN (blue), and HOECHST (gray) at D100. Scale bar 30 µm, 10 µm. (G) Box plots representing the amount of GABAergic synaptic proteins as ISD for each FOV: VGAT (left, CTRL *n* = 8/2 FOVs/batches; MUT *n* = 8/1 FOVs/batches; MUT BZ *n* = 8/2 FOVs/batches; *p < 0.05 **p < 0.01, one‐way ANOVA plus Sidak for multiple comparisons), GPHN (right, CTRL *n* = 8/2 FOVs/batches; MUT *n* = 8/1 FOVs/batches; NS one‐way ANOVA plus Sidak for multiple comparisons). (H) Scatter dot plot showing the quantitative real‐time PCR analysis of KCC2 (left) and NKCC1 (right) in D100 CTRL (white bars) and MUT (gray bars), and MUT BZ (black bars) organoids (CTRL *n* = 4/2, MUT *n* = 4/2, MUT BZ *n* = 4/2; batches/replicates (10 organoids each)). Gene expression is normalized to the housekeeping gene ATP5O (*p < 0.05, one‐way ANOVA with Tukey test for multiple comparison). (I) Representative traces of spontaneous calcium oscillations recorded from untreated MUT (top) and BZ‐treated MUT (bottom) D100 cortical organoids. Dot plots representing (J) the number of active neurons, (K) the frequency, and (L) the synchronicity of spontaneous calcium oscillations (CTRL, empty circles *n* = 47/4 FOVs/batches; MUT, gray circles *n* = 29/4 FOVs/batches; MUT BZ, black circles *n* = 29/4 FOVs/batches; *p < 0.05, **p < 0.01, ***p < 0.001, ****p < 0.0001, Kruskal Wallis test with Dunn's comparison). ANOVA, analysis of variance; CTRL, control; FOV, field of view; ISD, integrated signal density; MUT, mutant.

Based on the findings of BZ efficacy in normalizing mitochondrial function and promoting neuronal development in tau‐mutant cortical organoids, we extended our investigation to assess its potential in restoring synaptic connectivity, a crucial aspect of cortical development. Immunofluorescence analysis revealed that BZ treatment effectively rescued the SYN1 expression in tau‐mutant organoids, achieving levels comparable to those in control organoids at D100 (Figure 6A, B). Examining glutamatergic and GABAergic synaptic markers in neurons derived from organoid dissociation further supported the therapeutic potential of BZ. Specifically, for glutamatergic synapses, BZ treatment enhanced the expression of both pre‐synaptic (VGluT1, Figure 6C, D) and post‐synaptic (PSD95, Figure 6C, D) proteins, as well as their colocalization (Figure 6E), bringing the level of glutamatergic synapses in tau‐mutant neurons on par with control conditions. In the case of GABAergic synapses, BZ treatment led to an increased expression of the presynaptic marker (VGAT, Figure 6F, G) in tau‐mutant neurons without altering post‐synaptic Gephyrin levels (Figure 6F, G). To further investigate the effect of BZ on GABAergic transmission maturation, we performed RT‐qPCR for KCC2 and NKCC1, chloride transporters that regulate GABAergic function and its developmental switch. We observed that, at D100, NKCC1 expression was comparable in wild‐type (WT) and mutant organoids, but KCC2 was reduced in mutants compared to controls. After BZ treatment, KCC2 expression in mutants reached levels similar to controls, and NKCC1 decreased, indicating a shift toward a more mature, inhibitory GABAergic phenotype (Figure 6H), thus suggesting that BZ treatment not only rescues excitatory synapses but also promotes maturation of inhibitory circuits.

Furthermore, BZ treatment enhanced network functionality within tau‐mutant organoids. Calcium imaging recordings of spontaneous calcium transients in fast cells, most likely neurons (Figure 6I), showed that BZ treatment normalized the number of active neurons in tau‐mutant organoids to levels observed in controls (Figure 6J). This normalization was quantitative and qualitative, as the spontaneous activity patterns of active cells in BZ‐treated tau‐mutant organoids mirrored those in control organoids. Specifically, BZ treatment elevated the frequency (Figure 6K) and synchronicity (Figure 6L) of calcium events to levels similar to those observed in controls while maintaining peak amplitude characteristics (Figure S7F‐H). To confirm that the BZ effects observed in wide‐field calcium imaging were not confined to neurons belonging to superficial organoid layers, we performed complementary calcium imaging on 2D neuronal cultures derived from dissociated organoids. In these cultures, BZ treatment restored the amplitude and frequency of calcium transients to levels comparable to untreated controls, indicating that the observed rescue is not restricted to surface‐exposed cells (Figure S7D,E).

These data indicate that BZ, besides supporting cellular and mitochondrial health, restores neuronal network morphology and dynamics, suggesting a comprehensive therapeutic effect on tauopathy‐impacted neural systems.

## 4 DISCUSSION

Tauopathies, marked by abnormal tau aggregation, are a significant unmet clinical need linked to neurodegenerative diseases like AD and FTD. The roles of tau isoform dysregulation and hyperphosphorylation remain unclear. Effective treatments are lacking, highlighting the need for advanced in vitro models to study mechanisms and identify therapies. In this study, we found that the FTD‐linked IVS 10+16 MAPT mutation impaired neuronal development and network functionality in 3D cortical organoids and explored the therapeutic potential of bezafibrate to promote mitochondrial biogenesis and counteract the effects of tau mutation.

Specifically, tau‐mutant cortical organoids were characterized by: (i) tau isoform imbalance and increased levels of hyperphosphorylated tau, with impaired neuronal integrity; (ii) defective neuronal and glial maturation; (iii) synaptic and network dysfunction; and (iv) mitochondrial dysfunction. Treatment with BZ was mainly effective in normalizing: (i) mitochondrial parameters; (ii) neuronal integrity and synaptic maturation, and (iii) network functionality, highlighting its promise as a therapeutic strategy for tauopathies.

Isogenic hiPSC‐derived 3D cortical organoids were used to study the FTD‐linked IVS10+16 MAPT mutation. To enhance maturation, we modified an established protocol^25^ by supplementing the culture with heparin, ascorbic acid, BDNF, and GDNF.^31^, ^32^ Maturation was assessed in the control line by the upregulation of several genes (from D50 to D100), chiefly associated with synaptic transmission, protein binding, and acquisition of neuronal morphology, paralleled by a reduction in genes related to the cell cycle. We observed that the intronic tau mutation strongly impaired cortical organoid maturation.

We observed a downregulation of genes involved in excitatory and inhibitory synapses, neuronal morphology, and nervous system development, accompanied by an upregulation of genes associated with cell proliferation and stress, and a failure to downregulate cell cycle–related genes—a profile indicative of impaired neuronal maturation. Tau‐mutant organoids showed increased PAX6 levels, delayed neuronal and glial maturation, fragmented neurites, impaired neuronal subtype specification, and defective synaptic formation and network functionality. At D100, glutamatergic and GABAergic synapse‐related genes were significantly downregulated, confirmed by reduced SYN1 and SYNGR1 expression. Immunostaining of dissociated cortical neurons further revealed fewer glutamatergic and GABAergic synapses, consistent with tauopathy mouse models.^8^, ^10^ Time‐lapse imaging showed fewer active neurons with weaker spontaneous activity in tau‐mutant organoids.

These findings align with previous studies on 2D cortical neurons from the same iPSC lines, which showed impaired neuronal specification,^6^ altered excitability, reduced firing, and decreased calcium transients.^7^, ^40^ This supports a causal link between the IVS10+16 MAPT mutation and neuronal dysfunction. Additionally, our recent work with 2D and 3D iPSC‐derived retinal models revealed disrupted retinal cell differentiation, increased tau phosphorylation, and synaptic deficits,^8^ mirroring cortical dysfunction and emphasizing the systemic nature of tauopathies.

Our observations are also consistent with the recent examination of post‐mortem brain tissue from people who died with FTD with tau pathology caused by the MAPT intronic exon 10+16 mutation. Indeed, bulk tissue RNA sequencing revealed substantial downregulation of gene expression associated with synaptic function, further confirmed by histopathological analysis of the patient's cortex.^41^

We also found that the IVS 10+16 MAPT mutation caused a significant imbalance in tau isoforms, favoring 4R tau over 3R tau. This was evident from real‐time PCR, immunofluorescence, and Western blot analyses, indicating higher 4R tau expression in mutant organoids compared to controls, in line with data in 2D cortical cultures,^6^ and in retinal models.^8^ In addition, we report elevated levels of hyperphosphorylated tau at Ser202/Thr205 (AT8) and Thr181, revealed by Western blot and immunofluorescence analyses.

In line with this result, and with the impact of hyperphosphorylation in disrupting tau's role in microtubule stabilization,^42^ we observed a fragmented neurite morphology in tau‐mutant organoids, underscoring the mutation's impact on neuronal integrity and suggesting early‐stage disruptions in neuronal development. Our data, showing early deficits in synaptic maturation and neuronal differentiation, are in line with the notion that pathological tau, mislocalized into pre‐ and post‐synaptic neuronal compartments, may cause synaptic dysfunction.^43^, ^44^

Moreover, the observed impairment of cortical organoid maturation and function can be ascribed to the detrimental impact of pathological tau forms on mitochondrial function, which in turn may contribute to glial and neuronal damage and disease progression in AD and FTD.^13^, ^45^, ^46^, ^47^

Indeed, at D100, we found 17 “tau signature” genes^10^ differentially regulated in tau‐mutant organoids, encompassing synaptic vesicles, cytoskeletal structure, and mitochondrial and lysosome functions. This points to a broad impairment of neuronal network development and mitochondrial functions.

Specifically, when looking at all mitochondrial function‐related genes analyzed in the nanostring panel, we found 46 deregulated genes associated with autophagy, unfolded protein response, and oxidative stress.^48^, ^49^, ^50^, ^51^, ^52^, ^53^, ^54^ This aligns with another study that explored the impact of the MAPT IVS 10+16 mutation on cellular homeostasis, revealing deficits in lysosomal trafficking and acidity, which impair proteostasis in both neurons and astrocytes.^55^

Tau‐mutant organoids exhibited impaired mitochondrial biogenesis and function, with fewer and smaller mitochondria. At D100, PPARGC1A, a key regulator of mitochondrial biogenesis in both neurons and glial cells,^56^, ^57^ was significantly downregulated, particularly during the critical D50‐70 window, contributing to mitochondrial deficits essential for neuronal energy demands.

Mitochondrial dysfunction can promote tau abnormalities, enhancing phosphorylation and aggregation, further driving disease progression.^14^ Recent findings in the P301S tauopathy mouse model also link tau pathology to synaptic mitochondrial dysfunction, leading to bioenergetic deficits and synaptic loss.^43^ This suggests that targeting mitochondrial pathways could be a promising therapeutic strategy to mitigate synaptic impairment and cognitive decline in tauopathies like AD and FTD. Enhancing mitochondrial biogenesis and function could potentially alleviate some of the neurodegenerative processes associated with tau mutations that contribute to synaptic impairment and cognitive decline in AD and FTD.^12^, ^14^, ^47^, ^58^

Our study demonstrates the potential of BZ, a PPAR agonist, in mitigating mitochondrial dysfunction and improving neuronal integrity and function in tau‐mutant cortical organoids. MitoTracker labeling showed that BZ normalized mitochondrial parameters in tau‐mutant neurons, while RT‐PCR confirmed its role in restoring oxidative stress, unfolded protein response, and autophagy‐related gene expression to control levels, as reported in human iPSC‐derived neural progenitor cells.^59^

BZ treatment also promoted neuronal differentiation and maturation, evidenced by reduced PAX6 expression, fewer MAP2 fragments, and increased neurite length. Additionally, it restored synaptic connectivity and network function by prompting chloride equilibrium maturation^60^, ^61^ and rescuing synaptic marker expression and spontaneous calcium oscillations. These findings align with previous studies linking mitochondrial dysfunction to glutamatergic signaling deficits in MAPT 10+16 neurons,^62^ as well as research demonstrating mitochondrial‐targeted interventions in models of Leigh syndrome,^63^ and Huntington's disease.^64^ It should be noted, however, that calcium imaging and immunofluorescence do not directly inform on synaptic transmission or distinguish functional from silent synapses. To address this, future studies will incorporate electrophysiological techniques, such as patch‐clamp recordings to measure excitatory and inhibitory post‐synaptic currents,^65^ and multi‐electrode arrays to monitor extracellular activity across neuronal populations, enabling assessment of firing rates, burst dynamics, and network connectivity under varying conditions.

Although wide‐field calcium imaging primarily captures signals from superficial organoid layers, complementary experiments on dissociated neuronal cultures support the broader relevance of our findings. These neurons, representing all organoid layers, showed comparable functional impairments and responded similarly to bezafibrate, indicating that rescue effects are not confined to outermost regions. Moreover, immunostaining of dissociated cells confirmed bezafibrate impacts neuronal and mitochondrial phenotypes throughout the organoid.

BZ treatment not only enhanced neuronal maturation but also normalized 4R tau isoform expression and reduced phosphorylated tau to control levels. Studies in rats have shown that fibrillary tau and fetal tau share several phosphorylated sites.^66^ and that tau phosphorylation is high during early development and decreases with neurite stabilization and synaptogenesis.^67^ Thus, BZ's effects on neuronal maturation and synaptic activity likely result from reduced tau phosphorylation, mediated by improved mitochondrial function.^14^ This underscores the interconnected roles of mitochondrial health, tau regulation, and neuronal development.

Our findings may provide insight into mechanisms observed in rodent models of AD and FTD. In a rat model of sporadic AD induced by streptozotocin, BZ treatment improved cognitive function, reduced tau pathology, neuronal loss, and neuroinflammation.^39^ Similarly, in P301S transgenic mice, a familial FTD model, BZ decreased tau hyperphosphorylation, reduced microglial activation, and improved behavioral deficits.^38^

These results, alongside previous studies, suggest that BZ exerts multi‐targeted effects in AD and tauopathies, making it a promising therapeutic candidate. Additionally, our 3D in vitro disease model has proven valuable for studying tauopathies, testing new drugs, and repurposing existing ones. Its ability to replicate key aspects of tau‐related neurodegeneration makes it a reliable platform for evaluating therapeutics like BZ. Despite their utility, the model has limitations. iPSC‐derived organoids mainly reflect early brain development, limiting relevance to late‐onset diseases like Alzheimer's or familial FTD. Approaches such as prolonged culture, aging‐related stressors, or direct conversion may enhance maturity. Though not fully replicating the aged brain, these models reveal early vulnerabilities like mitochondrial dysfunction and synaptic disorganization. Additionally, the absence of microglia restricts studies on neuroinflammation and tau spread.^68^, ^69^ Future work will incorporate microglia‐like cells or immune co‐cultures to better model the aging brain's neuroimmune context.^17^, ^70^

## AUTHOR CONTRIBUTIONS

All authors have read and agreed to the published version of the manuscript. Federica Cordella performed organoids generation experiments, molecular, cellular and functional characterization; Lorenza Mautone performed molecular and immunofluorescence analysis on organoids; Lucrezia Tondo, Erika Parente, and Chiara D'Antoni performed immunofluorescence analysis and confocal acquisitions on neurons and astrocytes; Silvia Ghirga wrote the MATLAB code and performed quantification of confocal and calcium imaging acquisitions; Debora Salerno performed and analyzed nanostring experiments, Maria Anele Romeo and Mara Cirone designed, performed and analyzed Western blot experiments; Paola Bezzi and Silvia Di Angelantonio acquired funds and supervised the project; Silvia Di Angelantonio conceived the study, designed the experiments, interpreted the results, and wrote the manuscript with the help of Federica Cordella.

## CONFLICT OF INTEREST STATEMENT

The funders had no role in the design of the study; in the collection, analysis, or interpretation of data; in the writing of the manuscript; or in the decision to publish the results. S.D.A. is a scientific advisor of D‐Tails s.r.l. The remaining authors declare that the research was conducted in the absence of any commercial or financial relationships that could be construed as a potential conflict of interest. Author disclosures are available in the Supporting Information.

## ETHICS STATEMENT

All experiments were conducted following approval from the Ethics Committee for Transdisciplinary Research of Sapienza University (Protocol ID 5/2022). The iPSC lines used for this work were obtained from the European Bank for induced pluripotent Stem Cells (EBiSC), which adheres to strict privacy standards and GDPR compliance.

## Supporting information



Supporting Information

Supporting Information

Supporting Information

Supporting Information
