# Supplementary material for: Bezafibrate treatment rescues neurodevelopmental and neurodegenerative defects in 3D cortical organoid model of MAPT frontotemporal dementia
Source: Alzheimers Dement. 2025 Aug 14;21(8):e70419. doi: 10.1002/alz.70419 (PMC12351496; doi:10.1002/alz.70419)
Supplement: Supplementary file 3 — Supporting Information [file ALZ-21-e70419-s004.pdf]

Bezafibrate treatment rescues neurodevelopmental and neurodegenerative defects in 3D cortical organoid model of MAPT frontotemporal dementia

Federica Cordella[1,2]*, Lorenza Mautone[1,2]*, Debora Salerno[3], Lucrezia Tondo[1], Silvia Ghirga[2], Chiara D'Antoni[1,2], Erika Parente[1], Maria Anele Romeo[4], Mara Cirone[4], Paola Bezzi[1,5], Silvia Di Angelantonio[1,2,6].

SUPPORTING INFORMATION

Supplementary Table S1: Primary Antibodies for IF
Supplementary Table S2: Secondary Antibodies for IF
Supplementary Table S3: Primary and Secondary Antibodies for WB
Supplementary Table S4: Primers Sequences
Supplementary Figure S1: Methods for 3D and 2D cultures
Supplementary Figure S2: Housekeeping gene expression
Supplementary Figure S3: Heatmaps Nanostring D50-D100
Supplementary Table S5: DE genes D50 CTRL vs D50 MUT
Supplementary Table S6: DE genes D100 CTRL vs D100 MUT
Supplementary Figure S4: Heatmap and Gene Ontology Nanostring CTRL D50-D100
Supplementary Table S7: DE genes D50-D100 CTRL
Supplementary Figure S5: Heatmap and Gene Ontology Nanostring MUT D50-D100
Supplementary Table S8: DE genes D50-D100 MUT
Supplementary Figure S6: Gene expression of cell cycle-related genes and MAPT
Supplementary Figure S7: Calcium imaging analysis of organoids and dissociated neurons
Supplementary Figure S8: Gene expression of 4R/3R ratio, WB t-tau in BZ treated organoids

Supplementary Table S1

Primary Antibodies for IF	Cat. #	Provider	Final Dilution
mouse anti-PAX6	MA1-109	Invitrogen	1:100
chicken anti-GFAP	173006	Synaptic System	1:300
chicken anti-MAP2	ab5392	Abcam	1:2000
rabbit anti-TBR1	20932-1-AP	Proteintech	1:150
rabbit anti-β-TUBULIN III (TUJ1)	T2200	Sigma Aldrich	1:1000
rat anti-CTIP2	ab18465	Abcam	1:200
rabbit anti-synapsin 1	D12G5	Cell signaling	1:500
rabbit anti-SYNGR1	PA556226	Invitrogen	1:200
mouse anti-4R Tau	MMS-5020	Biolegend	1:200
mouse anti-AT8	MN1020	Invitrogen	1:200
anti phospho Tau181	MM0194	Medimabs	1:500
mouse anti-VGluT1	135303	Synaptic Systems	1:250
rabbit anti-PSD95	3450	Cell Signaling	1:250
mouse anti-gephyrin	147021	Synaptic System	1:250
rabbit anti-VGAT	131003	Synaptic system	1:300

Supplementary Table S2

Secondary Antibodies for IF	Cat. #	Provider	Final Dilution
AlexaFluor goat-anti rabbit PLUS 647	A32733	Invitrogen	1:500 (3D) – 1:750 (2D)
AlexaFluor goat-anti chicken PLUS 647	A32933	Invitrogen	1:500 (3D) – 1:750 (2D)
AlexaFluor goat-anti mouse PLUS 647	A32933	Invitrogen	1:500 (3D) – 1:750 (2D)
AlexaFluor goat-anti mouse PLUS 594	A32742	Invitrogen	1:500 (3D) – 1:750 (2D)
AlexaFluor goat-anti chicken 594	A32759	Invitrogen	1:500 (3D) – 1:750 (2D)
AlexaFluor goat-anti rat PLUS 594	A48264	Invitrogen	1:500 (3D) – 1:750 (2D)
AlexaFluor goat-anti rabbit PLUS 594	A32740	Invitrogen	1:500 (3D) – 1:750 (2D)
AlexaFluor goat-anti rabbit 488	A11008	Invitrogen	1:500 (3D) – 1:750 (2D)
AlexaFluor goat-anti rat 488	A11006	Invitrogen	1:500 (3D) – 1:750 (2D)
AlexaFluor goat-anti mouse PLUS 488	A32723	Invitrogen	1:500 (3D) – 1:750 (2D)

Supplementary Table S3

Antibodies for WB	Cat. #	Provider	Final Dilution
mouse anti-4R Tau	MMS-5020	Biolegend	1:500
mouse anti-AT8	MN1020	Invitrogen	1:250
rabbit anti-tau	D1M9X	Cell Signaling	1:500
mouse anti-β-actin	SAB4701038	Sigma Aldrich	1:10000
goat anti-mouse IgG-HRP	NC9462092	Bethyl Laboratories	1:20000
goat anti-rabbit IgG-HRP	NC0329705	Bethyl Laboratories	1:20000

Supplementary Table S4

Primers	Forward	Reverse
GAPDH	GGC CAT CCA CAG TCT TCT G	TCA TCA GCA ATG CCT CCT G
ATP5O	ACTCGGGTTTGACCTACAGC	GGTACTGAAGCATCGCACCT
TUJ1	TTT GGA CAT CTC TTC AGG CC	TTT CAC ACT CCT TCC GCA C
MAP2	TTCCTCCATTCTCCCTCCTCGG	TCTTCCCTGCTCTGCGAATTGG
RPLP0	CCTCATATCCGGGGGAATGTG	GCAGCAGCTGGCACCTTATTG
4R Tau	AGGCGGGAAGGTGCAGATAA	CTTTGAGCCACACTTGGACTGG
3R/4R Tau	TGAGAACCTGAAGCACCAGC	ATGAGCCACACTTGGAGGTC
XBP1 (PbD118640872c1)	CCCTCCAGAACATCTCCCCAT	ACATGACTGGGTCCAAGTTGT
CAST (Pb298919187c2)	CAAGCCGGGTGACAAGAAAAA	CCCGATGGTTTATCCGGTTTAG
PINK1 (Pb112382374c3)	CCCAAGCAACTAGCCCCTC	GGCAGCACATCAGGGTAGTC
CCNA2 (Pb166197663c1)	CGCTGGCGGTACTGAAGTC	GAGGAACGGTGACATGCTCAT
ACTB	TCATGAAGTGTGACGTGGACATC	CAGGAGGAGCAATGATCTTGATCT
NKCC1	ATTTGTAAGAGGAGGAGGAGCA	CCTAAAAGAATCACGACTGTAATGG
KCC2	TGGCACTCCTATCGACATGGAC	AGAGCGGTTAGAACCAGCCATG
GFAP	GATCAACTCACCGCCAACAG	ATAGGCAGCCAGGTTGTTCT
PPARGC1A	CCAGAGTCACCAAATGACCC	CCACAGTCTTGCAAGAGGACT
GS	TGGGAGCAGACAGAGCCTAT	TCCCAGGAATGGGCTTAGGA
PCNA (Pb33239449c1)	CCTGCTGGGATATTAGCTCCA	CAGCGGTAGGTGTCGAAGC

Supplementary Figure S1.

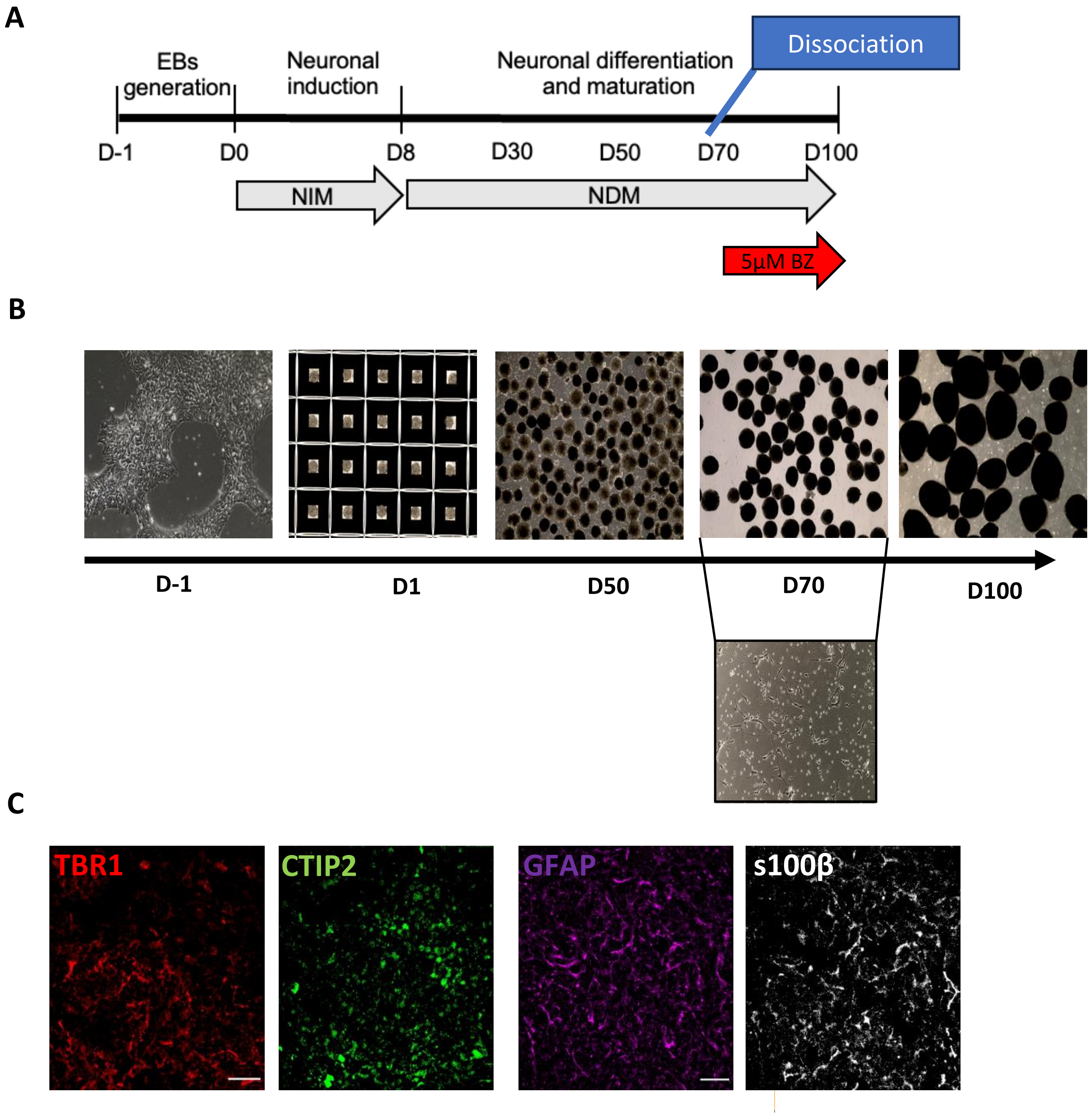

Figure S1. A) Representative scheme of differentiation protocol for hiPSC-derived cortical organoids and organoid dissociation at D70. **B)** Representative images of CTRL-derived cortical organoids development from D-1 to D100 of the differentiation protocol and 2D cortical cultures derived from organoid dissociation at D70. **C)** Representative images of CTRL-derived cortical organoids at D100 immunolabeled for neuronal and astrocytic markers. TBR1 (red), CTIP2 (green) (scale bar 30um); GFAP (magenta), S100b (grey) (Scale bar 30um).

Supplementary Figure S2.

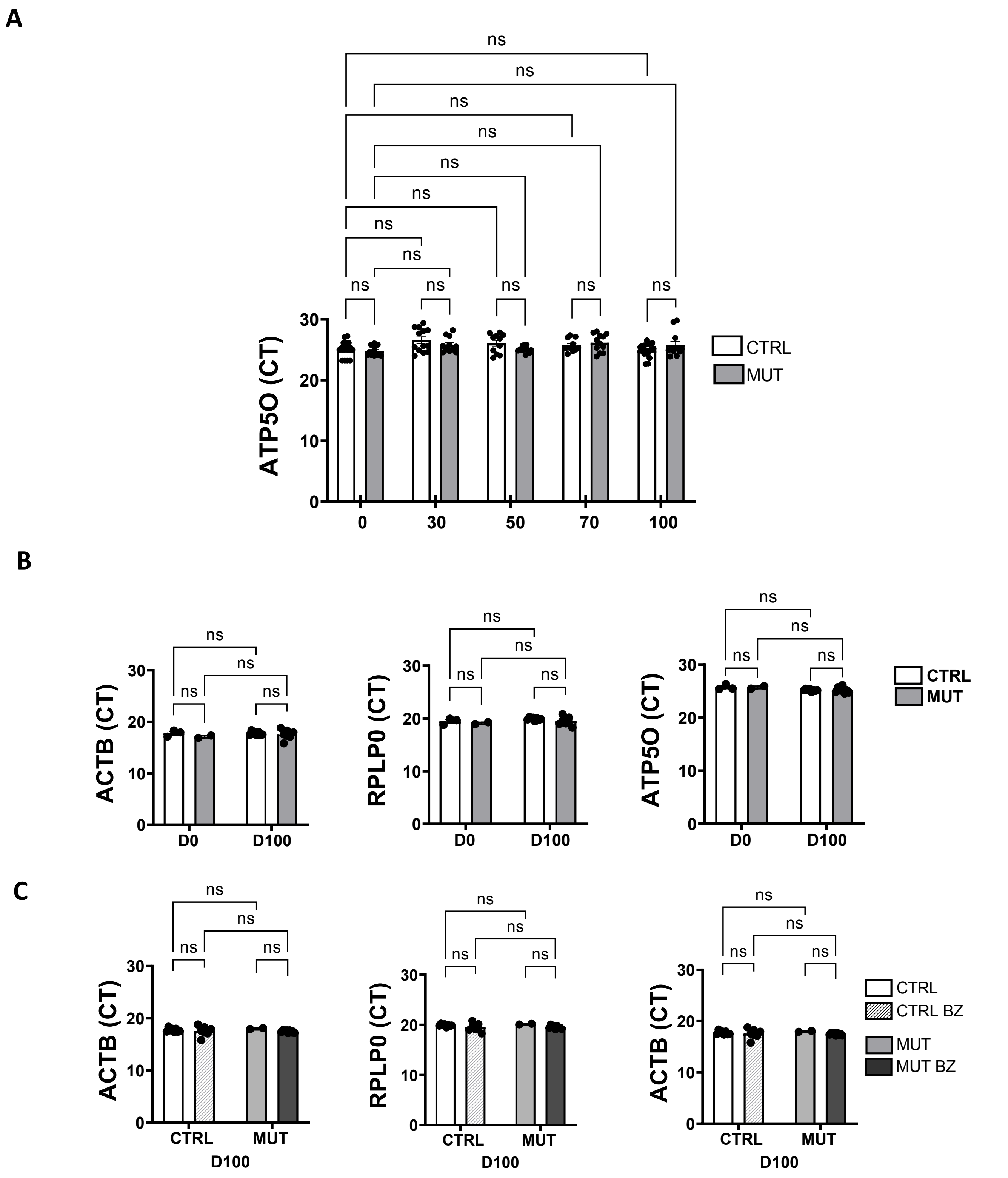

Figure S2. A) Scatter Dot plot reporting the Ct value of ATP5O on control (CTRL, white bars) and tau-mutant (MUT, grey bars) cortical organoids from D0 to D100. (CTRL n= 3/2, MUT n=4/2; replicates/batches); **B)** Scatter Dot plot reporting the Ct value of ACTB (left), RPLP0 (middle) and ATP5O (right) on control (CTRL, white bars) and tau-mutant (MUT, grey bars) cortical organoids from D0 to D100. (CTRL n= 3/2, MUT n=4/2; replicates/batches); **C)** Scatter Dot plot reporting the Ct value of ACTB (left), RPLP0 (middle) and ATP5O (right) on control (CTRL, white bars. CTRL BZ, white striped bars) and tau-mutant (MUT, grey bars. MUT BZ, dark grey bars) D100 cortical organoids (CTRL n= 3/2, MUT n=4/2; replicates/batches). One–way ANOVA with Tukey test for multiple comparison.

Supplementary Figure S3.

A

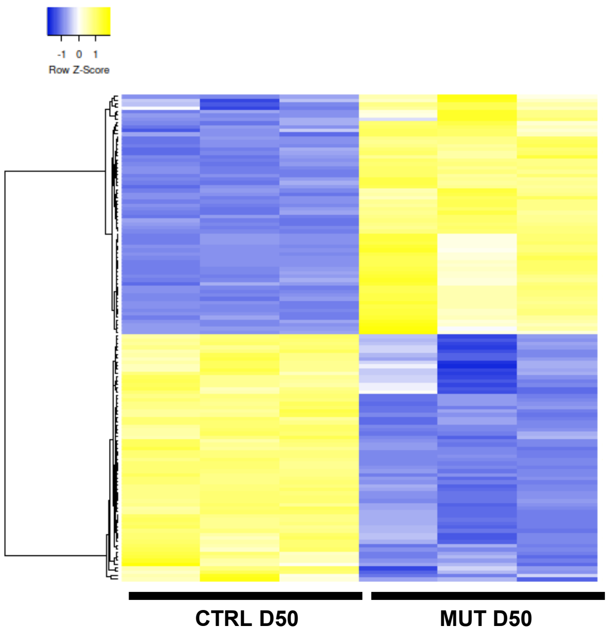

B

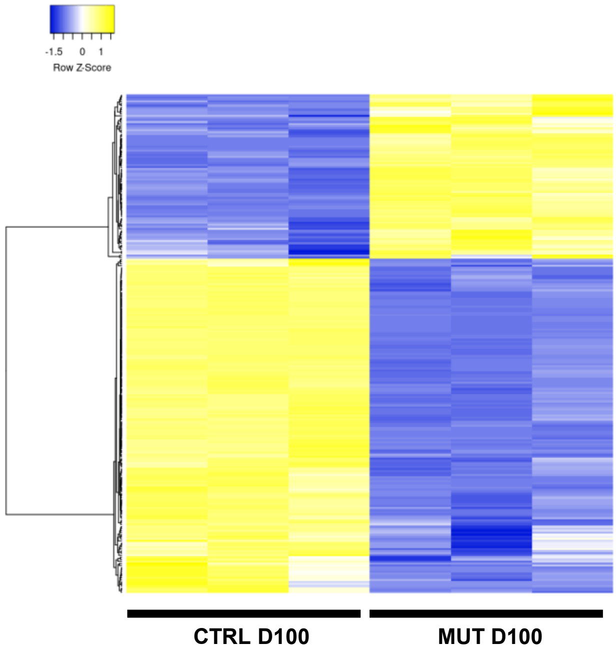

Figure S3. Heat map of unsupervised hierarchical clustering of: **A)** 130 differentially expressed (DE) genes between D50 CTRL and D50 MUT (64 genes up- and 66 genes down-regulated in D50 MUT) and **B)** 243 DE genes between D100 CTRL and D100 MUT (80 genes up- and 163 genes down-regulated in D50 MUT). Average linkege and Pearson Distance Measurement Methods were performed for Hierarchical cluster using Heatmapper tools (http://www.heatmapper.ca) . The significant DE genes are selected by multiple t-test performed with Graph Pad Prism 6, with p value ≤ 0.05 and a fold change cut-off values of ≥1.5 or ≤0.66 for upmodulated and downregulated genes respectively.

Supplementary Table S5

NAME	IPS d50 _1	IPS d50 _2	IPS d50 _3	IVS d50 _1	IVS d50 _2	IVS d50 _3
ABAT	3857.39	3844.04	3785.12	2652.00	2240.60	2262.43
ACHE	1275.57	1264.85	1250.12	650.52	684.25	638.12
ADCYAP1	137.12	94.14	129.80	25.61	19.82	22.64
AKT3	2878.61	2825.40	2769.11	6357.90	6491.78	6394.66
APOE	39.69	38.62	37.09	334.22	128.42	199.50
ARHGEF10	147.94	167.76	152.98	290.68	306.67	285.10
ATP6V0D1	3028.36	2748.16	2728.16	2028.38	1435.59	1606.62
ATP8A2	1990.04	2102.46	2121.64	1349.69	946.84	1166.59
B4GALT6	609.82	597.43	586.43	1061.57	906.59	959.31
C6	17.14	14.69	19.65	43.54	42.17	53.06
C9orf72	1032.91	1123.64	1135.77	1703.12	1730.76	1837.25
CALB1	1403.67	1218.99	1162.81	808.02	170.58	409.61
CAMK2D	1843.90	2033.66	1989.52	3576.56	2704.43	3292.48
CAMK2G	421.28	456.22	485.98	723.51	663.17	723.72
CASP3	1312.56	1250.37	1296.47	1910.57	2089.18	1949.74
CAST	209.29	220.87	244.92	449.47	329.67	381.32
CCNH	782.12	872.60	851.44	1376.58	1381.92	1453.81
CD9	16.37	14.69	19.65	78.11	49.83	62.26
CDK5	697.33	628.81	633.56	432.82	417.84	393.34
CDKN1A	1649.95	1599.17	1668.88	911.75	841.42	857.43
CHRNB2	783.93	819.50	769.54	487.89	379.50	461.26
CNTNAP1	616.14	726.57	665.24	452.03	381.42	440.04
CNTNAP2	973.37	1071.75	1033.01	2066.80	1548.67	1973.08
CRH	94.72	71.21	70.31	558.32	530.92	492.39
DDC	123.59	103.80	90.40	39.70	55.58	34.67
DGKB	726.19	833.98	836.76	340.62	195.50	242.66
DLX1	3834.84	3630.42	3785.89	997.54	1368.51	1146.78
DLX2	2358.10	2358.32	2229.81	513.50	898.92	695.43
DRD2	170.50	166.56	168.43	47.38	46.00	39.62
DRD4	22.55	21.72	34.00	90.92	74.75	75.70
EFNA1	16.37	14.69	19.65	55.06	53.67	50.94
EGF	16.37	18.10	19.65	60.19	70.92	62.26
EGFL7	30.67	14.69	19.65	39.70	46.00	36.79
EGFR	115.47	126.73	125.17	197.20	201.25	175.45
EGR1	94.72	91.73	102.76	40.98	49.83	48.11
EMP2	85.70	49.48	64.13	142.14	157.17	132.29
EPHA3	548.48	685.53	666.78	3187.27	2125.59	3003.14
FAS	209.29	245.01	224.84	176.71	92.00	149.27
FGF12	589.07	523.80	601.11	396.97	300.92	349.48
FGF14	3610.21	3549.56	3724.08	2538.04	1539.09	2142.17
FN1	478.11	526.22	555.52	284.28	230.00	261.05
FRMPD4	200.27	203.97	213.25	47.38	67.08	53.06
GABRA1	259.81	296.90	294.37	140.86	143.75	152.10
GABRA4	165.99	176.21	203.20	683.81	483.00	633.88
GABRG2	392.41	377.77	390.18	608.26	576.92	594.26
GAD1	3974.66	4476.47	4614.92	1243.41	1259.26	1267.05
GAD2	739.72	903.98	851.44	413.62	552.00	489.56
GAL3ST1	26.16	27.76	35.54	48.66	115.00	96.21
GDNF	27.06	35.00	25.50	19.01	19.82	15.32

**GNAI1**	2721.64	2463.33	2572.86	1272.86	1082.92	1197.01
**GPR37**	99.23	85.69	108.94	299.65	228.08	256.81
**GRIA2**	2956.19	3463.86	3281.36	9068.81	8628.88	10127.18
**GRIA4**	2193.01	2352.29	2337.21	778.57	588.42	761.22
**GRIN2A**	127.20	118.28	100.44	60.19	67.08	59.43
**GRM1**	72.17	78.45	82.67	366.24	233.83	365.75
**GUCY1B3**	308.52	271.56	291.28	797.78	868.25	839.04
**HAP1**	317.54	343.97	347.68	103.72	84.33	120.97
**HCN1**	73.07	74.83	83.44	40.98	42.17	38.20
**HNRNPM**	7893.40	7399.63	7636.68	4780.27	3718.35	4101.81
**IDH1**	2553.85	2524.88	2450.79	1385.55	1217.09	1279.07
**INHBB**	25.26	24.14	29.36	55.06	63.25	53.77
**IPCEF1**	158.77	164.14	165.34	357.27	245.33	311.28
**ITGA5**	27.06	25.35	27.81	97.32	51.75	55.18
**KCNA1**	23.45	18.10	19.65	49.94	86.25	66.50
**KIAA1161**	324.76	301.73	284.33	505.81	521.34	505.83
**LAMA2**	18.94	24.14	19.65	60.19	44.08	48.11
**LOX**	51.42	25.35	50.99	72.99	103.50	72.87
**MAPKAPK2**	451.05	432.08	441.94	781.13	596.09	622.56
**MGMT**	16.37	14.69	21.63	87.08	70.92	67.92
**MMP16**	2183.99	2569.54	2323.30	1379.14	975.59	1165.17
**MYD88**	33.38	42.24	30.13	19.01	19.82	25.47
**NAGLU**	58.64	55.52	47.90	107.57	105.42	89.85
**NEFH**	78.48	77.24	84.99	19.01	19.82	28.30
**NEFL**	12890.14	11903.86	11811.21	4921.13	5293.86	5064.65
**NEGR1**	3594.88	3494.04	3574.96	1863.19	1512.26	1762.97
**NFE2L2**	480.82	477.94	465.90	962.97	701.50	858.14
**NGFR**	298.60	292.07	276.60	117.81	105.42	99.75
**NKX6-2**	79.39	56.73	51.77	19.01	19.82	15.32
**NLGN4X**	1475.84	1535.20	1479.59	2412.54	2238.68	2384.82
**NOTCH1**	849.78	957.09	895.48	1253.65	1657.93	1452.40
**NOTCH3**	566.52	491.22	465.90	996.26	1042.67	951.52
**NOVA1**	6173.09	6560.82	6729.62	4613.80	3413.60	4294.24
**NR4A2**	200.27	176.21	195.48	143.42	63.25	75.70
**NRG1**	1143.87	1188.82	1236.98	489.17	318.17	418.10
**NSF**	1699.56	1589.52	1582.35	1143.52	931.50	1008.12
**NTF3**	16.37	14.69	19.65	76.83	44.08	53.06
**NTNG1**	123.59	196.73	181.57	1083.34	734.09	993.97
**NTRK1**	821.82	811.05	720.86	44.82	19.82	28.30
**NTS**	786.63	784.50	939.52	332.94	429.34	358.68
**OLIG2**	414.97	434.49	368.54	48.66	126.50	76.40
**PCSK2**	866.02	884.67	812.04	2251.19	1602.34	2126.60
**PDGFRB**	42.40	44.66	34.00	186.96	130.33	145.03
**PIK3R1**	1713.09	1757.28	1755.42	810.58	678.50	793.76
**PLA2G4F**	44.20	47.07	40.18	30.73	19.82	28.30
**PLCB1**	277.85	329.49	319.10	541.67	421.67	487.43
**PLS1**	35.18	36.21	39.40	66.59	53.67	61.55
**POLR2L**	2763.14	2295.56	2542.73	1908.01	1176.84	1257.14
**PRKACA**	3229.53	3002.82	3024.08	1928.50	1600.43	1659.68
**PRKACB**	7919.56	8032.06	8019.14	5100.40	4042.27	4575.80

**PRKCB**	978.78	867.78	944.93	514.78	488.75	503.71
**PRKCG**	82.09	83.28	76.49	125.49	145.67	132.29
**PTPRR**	95.62	109.83	112.80	190.80	172.50	171.20
**RAB3C**	3861.90	3689.56	3687.00	2462.48	2048.93	2126.60
**RASGRP1**	204.78	187.07	147.57	877.17	582.67	745.65
**RET**	253.49	287.25	302.87	185.68	101.58	140.78
**RRAS**	16.37	14.69	19.65	35.86	38.33	29.01
**SCN1A**	508.79	558.80	521.53	404.65	266.42	367.17
**SERPINB6**	420.38	504.49	450.44	325.26	210.83	289.35
**SHH**	650.42	708.46	649.01	72.99	76.67	68.62
**SLA**	16.37	14.69	19.65	53.78	59.42	54.47
**SLC17A6**	851.58	741.05	780.36	2742.92	2127.51	2479.62
**SLC18A3**	38.79	28.97	25.50	19.01	19.82	15.32
**SLC1A2**	931.87	918.47	881.57	1818.37	1539.09	1708.50
**SLC32A1**	371.67	308.97	356.18	128.05	172.50	133.00
**SLC4A10**	341.90	453.80	399.45	1430.37	898.92	1264.92
**SORL1**	221.02	232.94	256.51	376.48	360.34	379.90
**STAMBPL1**	340.99	341.56	385.54	1413.72	1063.76	1256.44
**SYT1**	6426.58	5947.71	5968.57	4420.43	2980.43	3757.28
**SYT7**	558.40	562.43	615.79	1274.14	1188.34	1197.72
**TBR1**	165.08	179.83	189.29	5013.33	2995.77	4062.19
**TCIRG1**	64.05	72.42	80.35	26.89	19.82	36.08
**TENM2**	870.53	957.09	912.48	548.07	308.58	489.56
**TF**	29.77	35.00	40.95	60.19	59.42	49.52
**TH**	304.01	339.14	352.32	124.21	226.17	184.65
**THY1**	1499.29	1357.79	1476.50	959.13	795.42	880.78
**TNC**	109.15	125.52	113.58	244.58	322.00	269.54
**TNFRSF10I**	45.11	54.31	50.99	19.01	19.82	25.47
**TSPO**	16.37	14.69	19.65	42.26	63.25	36.08
**UGT8**	61.34	55.52	61.81	151.10	195.50	194.55
**XK**	135.32	115.86	137.53	58.90	19.82	36.08

Supplementary Table S6

NAME	IPS d100 _1	IPS d100 _2	IPS d100 _3	IVS d100 _1	IVS d100 _2	IVS d100 _3
NTRK1	247.37	211.78	146.99	1.72	1	1
SLC32A1	718.68	684.43	808.95	20.41	1	3.14
NKX6-2	122.49	97.9	56.35	1.72	1	1
SLC18A3	61.67	52.55	17.9	1	1	1
ADCYAP1	191.99	177.51	177.21	5.46	1	6.38
GAD2	1814.41	1844.38	2478.97	91.45	24.7	32.32
GAD1	11286.15	11290.31	12911.05	418.6	127.08	323.23
MMP2	421.13	400.23	100.3	8.26	1	15.3
DDC	73.62	87.82	45.36	1	4.59	1
TLR4	70.36	73.71	56.35	4.52	1	1
TH	618.77	682.41	627.67	26.02	10.08	43.66
AQP4	157.24	174.49	94.8	10.13	5.51	4.76
DLX1	3953.75	3826.69	4772.48	403.65	133.48	80.94
NGFR	413.52	431.47	325.53	12.94	17.39	28.26
DLX2	1879.57	1838.34	2083.44	195.21	84.12	33.13
FRMPD4	294.07	342.79	284.33	19.48	7.34	27.45
TENM2	1360.48	1366.69	1333.58	90.52	53.04	99.57
DRD2	284.29	279.3	218.41	18.54	15.56	12.87
OLIG2	263.66	230.93	177.21	24.15	21.05	1
SHH	335.33	294.42	443.64	46.59	26.53	2.33
NEFL	23874.6	25396.2	24735.72	1854.33	1277.93	2573.54
FGF14	5010.39	4967.49	6483.7	438.23	253.23	581.73
GABRA1	648.09	649.16	602.95	57.8	33.84	59.06
MBP	135.52	115.03	67.34	7.33	1	18.54
RET	282.12	264.18	309.05	30.7	4.59	42.04
PTPRR	296.24	298.45	325.53	33.5	19.22	33.13
HAP1	543.84	615.9	646.9	60.61	53.04	81.75
RAB3C	5650.02	5742.48	5725.6	693.41	538.43	623.86
CALB1	2191.24	2185.01	2316.91	287.74	90.52	369.42
GRIA4	3629.05	3755.13	3838.6	407.39	431.48	469.9
ACHE	2914.49	2984.18	3179.38	336.35	291.62	469.09
SLC12A5	1248.63	1224.6	1251.18	149.4	137.14	188.71
PDE1B	219.14	208.76	177.21	28.83	4.59	52.57
DRD1	84.48	81.78	92.06	9.2	8.25	19.35
NEFH	178.96	189.61	174.46	26.96	23.79	26.64
XK	226.74	201.7	185.45	28.83	22.88	37.18
DBH	9.55	8.21	2.75	1	1	1
HCN1	287.55	293.41	270.59	41.91	28.36	58.25
CYP4X1	605.74	663.26	608.44	82.11	58.53	144.14
RYR3	464.56	492.95	449.13	80.24	50.3	102.81
GABRB2	1803.55	1902.83	1674.17	284	277.91	375.09
SORCS3	414.61	444.58	506.81	66.22	56.7	116.59
S100B	1103.11	940.4	564.49	218.57	111.54	265.69
NPY	887	779.16	449.13	136.32	164.56	183.04
CD8A	107.28	78.75	53.6	10.13	10.99	34.75
DGKB	1928.44	1820.2	2034	532.64	451.59	401.83
GRM5	1407.18	1410.03	1352.81	354.11	317.22	332.14
SCN1A	1402.83	1423.13	1487.4	321.39	285.22	441.54
RIMS1	905.46	896.06	883.12	207.36	186.5	286.76

**NOVA1**	5993.19	6335.05	7288.49	1771.14	1600.61	1651.37
**HMOX1**	99.68	69.68	111.28	26.96	6.42	42.04
**HLA-DRA**	5.2	3.17	2.75	1	1	1
**PRKCB**	1161.75	1175.22	1072.64	212.03	397.66	321.61
**CCND1**	816.41	847.69	723.8	270.92	116.11	273.8
**PTPRN2**	3404.26	3319.77	3385.39	924.29	863.85	1016.07
**FOS**	1079.22	1019.01	1163.28	251.29	248.66	435.05
**RYR1**	150.72	136.2	188.19	47.52	32.93	56.63
**ADCY8**	345.11	321.63	314.54	83.04	53.04	149
**EFNA5**	671.98	681.4	800.71	180.25	168.22	281.09
**GRIN2D**	644.83	627.99	715.56	198.01	190.16	231.66
**THY1**	3755.02	3907.31	3794.65	1165.44	1132.59	1302.12
**CACNB2**	604.65	695.51	674.36	199.88	155.42	276.23
**PRKACB**	10239.29	10335.94	11411.33	3245.19	3635.39	3452.76
**CX3CL1**	176.78	157.36	136	59.67	23.79	70.4
**PLXNC1**	1633.06	1627.71	1572.54	578.44	564.94	501.5
**NRG1**	1217.13	1172.19	1240.19	423.28	418.68	398.59
**SLC1A1**	1118.31	1163.12	1193.5	372.8	380.29	452.07
**OLFM3**	1104.19	1089.56	907.84	389.63	336.41	362.93
**FGF12**	1629.8	1574.3	1720.87	510.21	527.46	721.1
**NSF**	2549.61	2666.73	2531.15	895.31	902.24	995.81
**CADPS**	2328.07	2355.33	2341.63	858.85	761.47	961.77
**COL4A1**	919.58	846.68	608.44	304.57	302.59	273.8
**MAGEE1**	1269.26	1301.19	1306.11	474.69	477.18	507.98
**NEGR1**	5225.41	5074.32	5198.23	1587.93	1950.71	2387.97
**FN1**	867.46	747.92	677.11	326.07	226.72	328.9
**XBP1**	574.25	541.32	498.57	208.29	208.44	228.42
**GSN**	513.43	445.58	322.78	208.29	109.71	195.19
**TNR**	1203.02	1240.72	1198.99	451.32	572.25	490.16
**SYT13**	3545.43	3484.04	3434.83	1252.37	1485.43	1633.55
**PIK3R1**	2031.6	2072.14	2245.49	766.32	822.71	1092.24
**MAPK10**	4401.17	4492.83	4511.55	1753.38	1763.32	2178.9
**ATP8A2**	3138.2	3179.69	3127.19	1005.61	1395.85	1640.84
**UNC13A**	2157.58	2251.53	2388.32	928.96	914.12	1127.89
**PLA2G16**	810.99	850.71	792.47	419.54	183.76	474.76
**INA**	13472.19	13702.93	13718.59	5517.48	5740.56	6723.29
**SCN2A**	3544.35	3579.78	3514.48	1468.29	1652.71	1800.48
**SNCB**	1032.52	1038.16	998.48	406.45	514.66	499.07
**MAPT**	3923.35	3780.33	3682.03	1650.56	1744.12	1875.84
**CACNB4**	786.01	806.37	773.25	343.83	348.29	413.17
**SERPINB6**	693.7	667.3	613.94	305.5	264.2	366.17
**CNTN1**	3898.37	4136.07	3764.44	1733.75	1968.08	1952.01
**ITPR2**	237.6	236.97	251.37	147.54	96	107.68
**PLXNB3**	90.99	73.71	70.08	37.24	23.79	52.57
**CHRNA7**	853.34	890.01	894.1	444.78	372.98	459.36
**PINK1**	1554.87	1521.89	1325.34	687.8	663.66	794.84
**GAL3ST1**	51.9	43.48	34.38	12	24.7	26.64
**GNG2**	10579.2	10273.45	10367.58	4803.36	5226.84	5385.42
**FAS**	200.68	181.55	185.45	124.17	73.15	84.18
**NRXN1**	1652.6	1576.31	2212.53	921.48	929.66	852.38

**STAT3**	1927.35	1833.3	1935.11	987.85	798.95	1069.55
**PLCB4**	1102.02	1052.27	1047.92	562.55	482.67	580.1
**CNTNAP1**	995.6	1085.52	1366.54	571.9	514.66	665.19
**NOL3**	205.02	227.9	240.38	127.91	88.69	126.31
**ATP6V0C**	9294.5	8822.25	9439.18	4460.32	4580.57	5130.16
**PPARGC1A**	644.83	714.66	718.31	382.15	207.52	503.93
**GALC**	471.08	496.98	451.88	242.88	266.94	238.95
**AMIGO1**	233.25	218.83	193.69	117.62	107.89	115.78
**ATP13A2**	1911.06	1849.42	1803.27	908.39	967.14	1071.17
**CACNA1C**	1363.74	1463.44	1448.94	694.34	643.55	929.36
**KCNB1**	429.81	415.35	468.36	223.25	224.89	259.21
**PLA2G4C**	336.42	348.84	278.83	154.08	176.44	188.71
**SOD2**	2578.93	2633.47	2915.7	1450.53	1409.56	1518.48
**CAST**	405.92	432.48	460.12	244.75	173.7	281.9
**SLC17A6**	848.99	946.45	833.67	433.56	278.82	714.62
**PRKACA**	3479.19	3684.59	3693.02	1897.33	1980.87	2048.44
**ATP6V0E2**	1851.33	1940.12	1882.92	1012.15	1036.61	1064.69
**UCHL1**	15510.54	15754.77	16756.47	8345.93	9026.75	9034.37
**GNPTG**	833.79	877.92	803.46	473.75	437.88	473.95
**UGCG**	1881.74	1858.49	1841.72	1028.04	1007.36	1071.17
**AMPH**	2412.78	2537.74	2465.23	1410.34	1213.95	1503.08
**PCSK2**	781.66	852.73	685.35	435.43	289.79	576.05
**SLC9A6**	1390.89	1478.56	1383.02	772.86	794.37	833.74
**NQO1**	491.71	479.85	484.84	310.18	159.99	353.21
**COL4A2**	663.29	700.55	531.53	368.13	392.17	319.99
**CAMK2B**	1057.5	1094.59	1130.32	544.79	561.28	764.86
**MMP24**	866.37	906.14	762.26	407.39	434.22	610.09
**ERLEC1**	2868.88	2933.79	2912.95	1634.67	1730.41	1635.98
**EFNB3**	2899.29	2891.47	2901.96	1899.19	1592.38	1541.17
**ATP6V1G2**	2316.13	2349.28	2281.2	1287.89	1420.53	1327.24
**MYC**	411.35	390.16	416.17	216.7	221.23	273.8
**ATP6V1A**	6560.06	6655.52	6719.92	3848.08	3860.26	4024.05
**ARC**	151.81	160.38	144.24	87.71	101.49	82.56
**INPP5F**	4583.61	4787.1	4665.36	2659.12	2763.34	2985.19
**SYT1**	10729.06	10449.82	10510.4	5631.52	6854.85	6584.72
**GNAI1**	3376.02	3522.34	3308.48	1815.07	2149.07	2184.58
**L1CAM**	4473.93	4787.1	5126.81	2657.25	2871.21	3182.91
**PRNP**	5133.11	5381.69	5069.13	3176.95	2388.56	3886.29
**ATP6V0D1**	4180.72	4166.31	4206.66	2506.76	2444.32	2691.04
**POLR2K**	2349.79	2354.32	2336.14	1370.14	1595.13	1337.77
**CHMP2B**	1085.73	1159.09	1220.96	677.52	736.79	719.48
**SNAP91**	2989.42	3016.43	3393.63	1789.83	2022.01	1998.2
**ABAT**	4226.33	4079.64	4365.97	2650.71	2679.25	2524.92
**CYCS**	959.76	1059.32	1067.15	602.74	614.3	703.28
**GABRG2**	877.23	874.9	715.56	520.49	479.92	540.4
**MMP16**	2008.8	2205.17	2303.17	1242.09	1338.26	1503.89
**MAP2K1**	1310.53	1383.83	1226.46	841.1	822.71	807
**GRIN1**	297.33	292.4	270.59	181.19	165.47	196
**GABRA4**	530.81	612.88	597.46	322.33	296.19	485.29
**GABRB3**	3042.63	3072.87	2869	1794.51	1845.59	2060.59

**TNFRSF1A**	309.27	327.67	289.82	206.42	136.22	248.68
**GNAO1**	5317.72	5530.84	4896.09	3151.71	3273.41	3647.24
**CDK5**	1034.69	979.71	979.25	629.85	619.78	666
**OPTN**	2114.14	2184	2196.05	1334.63	1333.69	1529.01
**NTS**	2904.72	3426.6	3357.92	2155.31	1553.08	2567.06
**ATP6V1H**	2631.05	2662.7	2704.2	1605.69	1772.46	1805.34
**STAMBPL1**	450.45	497.99	564.49	298.02	284.31	399.4
**APP**	15729.9	15920.05	15481.99	9745.21	9856.75	11019.71
**CDKN1A**	1488.62	1546.08	1407.74	1092.53	749.58	1049.29
**BACE1**	1964.27	1947.18	1808.76	1146.75	1249.6	1357.22
**NLGN4X**	2361.74	2275.71	2503.69	1681.41	1519.26	1486.06
**DDIT3**	1005.37	1159.09	1144.05	746.69	549.4	888.03
**BCAS2**	1961.02	1955.24	2075.2	1313.13	1382.14	1287.53
**PRKCE**	410.27	398.22	314.54	189.6	255.06	302.16
**IDE**	342.94	328.68	292.57	491.51	457.99	504.74
**GUSB**	595.97	659.23	633.16	984.11	960.74	936.65
**CCNH**	870.71	880.94	863.89	1299.11	1364.77	1347.5
**EPHA4**	1368.08	1363.67	1435.21	2307.67	2272.47	1845.04
**RAF1**	1652.6	1728.49	1654.95	2530.13	2717.64	2569.49
**SGPL1**	508	429.46	451.88	690.61	771.52	697.6
**ARHGEF10**	201.76	176.51	116.78	285.87	248.66	237.33
**STX2**	997.77	1011.96	1097.36	1652.43	1577.76	1618.15
**DLG3**	565.56	604.81	553.51	855.12	904.07	938.27
**CNTN4**	270.18	265.19	254.11	363.46	383.94	493.4
**GNAI2**	2865.62	2925.73	2926.68	4696.8	4946.21	4173.15
**LSM2**	733.88	726.75	597.46	1101.88	1225.83	949.62
**MUTYH**	134.43	105.96	97.55	171.84	191.98	175.75
**NES**	4090.58	3811.57	3629.85	6583.06	6403.28	5718.47
**TCERG1**	1224.74	1291.11	1358.3	2076.79	2127.13	2084.09
**GUCY1B3**	738.23	699.54	515.05	1019.63	1114.31	1050.1
**SLC1A2**	1279.03	1343.52	1144.05	2184.28	2115.25	1862.06
**CDK5RAP3**	1553.78	1709.34	1696.15	2704.92	2522.94	2975.47
**C5**	105.11	100.92	75.58	155.01	150.85	161.97
**FAM126A**	733.88	682.41	635.91	1024.3	1115.22	1277.81
**TAF6L**	143.12	186.58	122.27	251.29	245	261.64
**ACTN1**	410.27	493.96	468.36	810.25	680.11	812.67
**SNRPA**	788.18	825.52	850.15	1406.6	1454.35	1302.12
**SNCAIP**	380.95	312.56	309.05	607.42	637.15	472.33
**TAF4**	611.17	566.52	506.81	1000	996.39	961.77
**TP53**	439.59	456.67	353	785.01	788.89	634.4
**IKBKB**	272.35	244.03	240.38	462.53	455.24	424.52
**DOT1L**	1122.66	1200.41	1286.88	2134.74	2118.9	2164.32
**HDAC1**	654.61	602.8	622.18	1165.44	1190.18	1069.55
**NFE2L2**	701.3	646.13	605.7	1250.5	1205.72	1175.7
**MAN2B1**	174.61	208.76	160.73	340.09	324.53	362.12
**DLGAP1**	1077.05	1209.48	946.29	1849.65	2203.91	2074.37
**KATNA1**	442.84	384.11	347.5	800.9	780.66	713.81
**PQBP1**	1211.7	1272.97	1330.83	2385.25	2624.4	2554.09
**EPHA5**	805.56	748.93	641.4	1385.1	1783.43	1268.89
**GPR37**	175.7	175.5	86.56	245.68	315.39	325.66

**PCNA**	272.35	272.25	256.86	617.7	624.35	389.67
**MAPKAPK2**	384.2	450.62	388.7	824.27	826.37	854
**MTA2**	1319.21	1343.52	1410.49	2826.43	2978.16	2588.12
**IPCEF1**	155.07	157.36	108.54	302.7	281.57	284.33
**KIAA1161**	464.56	448.61	399.69	844.83	1033.87	832.93
**LOX**	52.99	48.52	15.15	92.39	79.55	72.02
**AGER**	99.68	118.06	67.34	219.51	200.21	205.73
**PLCB3**	102.94	92.86	116.78	221.38	237.69	226.8
**SLC4A10**	612.25	641.09	644.15	1282.28	1267.88	1702.42
**SP1**	144.21	133.17	103.04	321.39	309.9	263.26
**SOX9**	1606.99	1693.22	1578.04	4323.85	4018.4	3467.34
**BID**	185.47	177.51	146.99	395.24	405.88	440.73
**PARP1**	1450.62	1380.8	1333.58	3541.49	3559.52	3246.93
**CNTF**	100.77	97.9	64.59	206.42	239.52	229.23
**PHF19**	68.19	64.64	23.39	139.12	156.33	111.73
**INHBB**	60.59	68.67	34.38	171.84	147.19	116.59
**NOTCH3**	386.37	353.88	289.82	1013.08	946.12	827.26
**NAGLU**	63.84	60.61	39.87	148.47	132.57	165.21
**PRKCG**	51.9	53.56	53.6	142.86	132.57	164.4
**CASP7**	71.45	53.56	31.63	190.53	150.85	128.75
**NELL2**	3109.96	3053.72	3239.81	8658.13	10321.11	9820.4
**NOTCH1**	742.57	682.41	605.7	2474.05	2453.46	1771.3
**GRIA3**	1721.02	1684.15	1646.71	4983.76	6059.58	6160.1
**AKT3**	3013.31	3023.49	2706.94	9821.85	10960.98	9221.56
**CASP6**	243.03	213.79	199.18	771.93	831.85	741.36
**EMP2**	63.84	53.56	2.75	151.27	162.73	119.83
**GRIN3B**	1.09	1.01	2.75	7.33	7.34	5.57
**PLS1**	38.87	24.33	2.75	110.15	55.78	123.88
**EGF**	28.01	21.31	2.75	76.5	106.97	80.94
**CDK2**	71.45	54.56	12.4	291.48	268.77	192.76
**EPHA7**	550.35	640.09	498.57	3254.53	3759.71	3076.76
**HGF**	12.8	18.28	2.75	78.37	85.03	65.54
**ADRA2A**	97.51	80.77	26.14	343.83	617.95	843.46
**DRD4**	5.2	1.01	2.75	33.5	22.88	24.21
**GRIN2C**	12.8	1.01	2.75	45.65	45.73	60.68
**CA2**	157.24	166.43	141.5	1775.81	561.28	2174.85
**KCNJ10**	11.72	5.18	2.75	71.82	58.53	63.92
**NOSTRIN**	1.09	1.01	2.75	19.48	18.31	14.49
**MGMT**	1.94	1.15	2.75	33.5	23.79	16.11
**NTF3**	1.09	1.01	2.75	41.91	28.36	41.23
**TSPO**	3.03	3.17	2.75	59.67	96	65.54
**EFNA1**	1.09	1.01	2.75	65.28	97.83	123.07
**TBR1**	59.5	67.67	39.87	5189.4	6225.95	6626.04
**SLA**	1.09	1.01	2.75	860.72	1258.74	1202.44

Supplementary Figure S4.

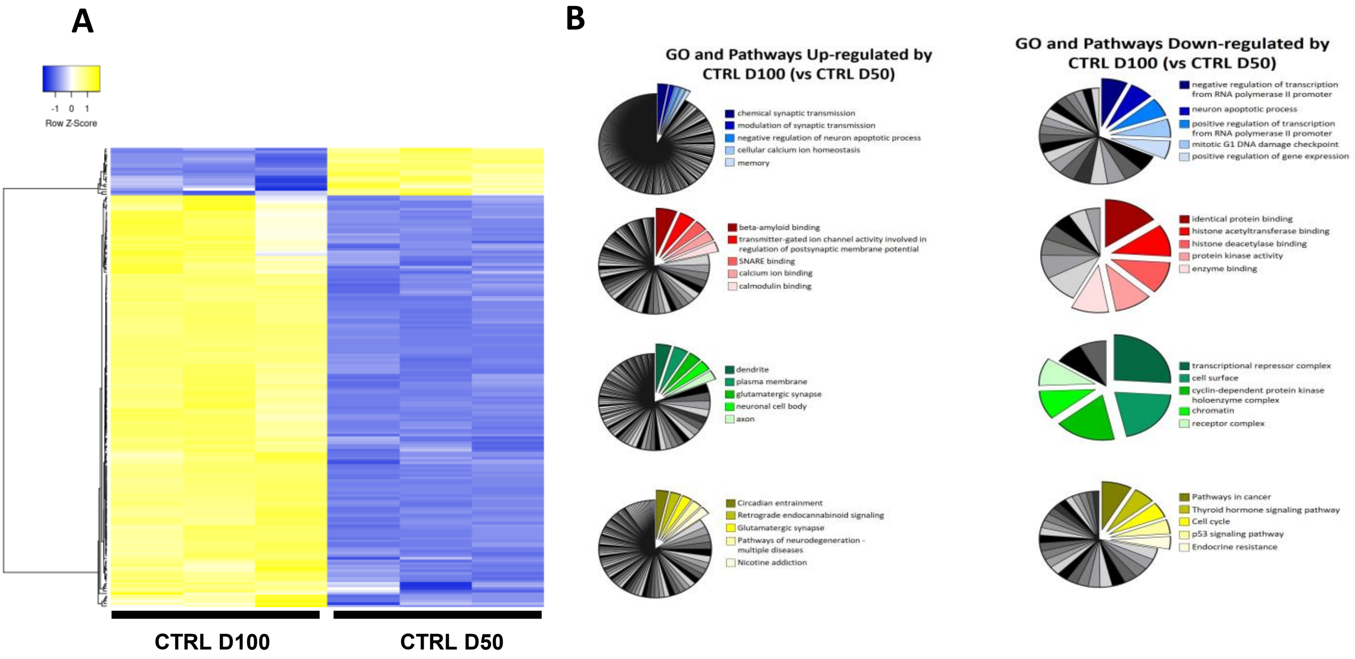

Figure S4. A) Heat map of unsupervised hierarchical clustering of 183 differentially expressed (DE) genes between D50 CTRL and D100 CTRL (164 genes up- and 19 genes down-regulated in D100 CTRL). Average linkege and Pearson Distance Measurement Methods were performed for Hierarchical cluster using Heatmapper tools (http://www.heatmapper.ca). The significant DE genes are selected by multiple t-test performed with Graph Pad Prism 6, with pvalue ≤ 0.05 and a fold change cut-off values of ≥1.5 or ≤0.66 for upmodulated and downregulated genes respectively. **B)** Gene Ontology (GO) and KEGG Pathways enrichment analysis of 183 down- and up-regulated genes by D100 CTRL (respect to D50 CTRL) have been analyzed by DAVID database. An overview of top 5 significantly enriched terms in KEGG pathways and three GO categories (biological process (BP), cellular component (CC), and molecular function (MF)) are showed. The cut-off of p-value was set to 0.05 and are selected only enriched terms in which are represented a minimum of 3 genes. The −log10 p-value associated with each category is represented by the size of the pie slice.

Supplementary Table S7

Probe Name	IPS d50 _1	IPS d50 _2	IPS d50 _3	IPS d100 _1	IPS d100 _2	IPS d100 _3
ACHE	1207.81	1199.61	1183.83	2656.07	2719.58	2897.47
ADCY5	559.67	613.33	615.74	915.24	897.43	919.96
ADCY8	122.39	122.26	159.50	314.51	293.11	286.65
ADCYAP1	117.21	73.50	109.87	174.96	161.77	161.49
ADORA1	138.81	106.00	110.61	243.25	223.31	299.17
AGER	214.86	231.38	221.71	90.84	107.59	61.37
AMIGO1	131.04	124.58	110.61	212.57	199.43	176.51
APOE	23.88	20.09	21.00	278.88	273.82	274.14
AQP4	10.91	1.16	1.00	143.30	159.02	86.40
ARC	102.52	60.73	89.14	138.35	146.16	131.45
ARHGAP44	284.00	302.20	312.07	521.35	553.02	549.49
ATP13A2	1014.23	1067.26	1030.51	1741.61	1685.44	1643.38
ATP2B3	128.44	167.53	128.39	242.26	264.64	234.09
ATP6V0C	5725.75	5402.20	5353.72	8470.39	8040.01	8602.24
ATP6V0E2	1206.08	1053.33	1057.17	1687.18	1768.10	1715.97
ATP6V1G2	1379.78	1364.46	1354.18	2110.76	2140.98	2078.93
B4GALT6	570.04	557.61	547.60	891.49	902.94	819.83
BACE1	1060.03	1125.31	1030.51	1790.11	1774.53	1648.39
BCHE	246.84	205.84	206.16	709.39	644.86	351.74
BDNF	51.53	47.96	57.29	103.71	107.59	101.42
BNIP3	685.84	629.59	632.78	962.74	1011.32	1035.10
CA2	103.38	68.85	66.92	143.30	151.67	128.95
CACNA1B	432.64	527.42	526.86	724.23	784.47	804.81
CACNB2	344.49	364.89	377.25	551.04	633.84	614.57
CADM3	1868.04	1872.95	1812.64	2738.21	2832.55	2957.55
CALB1	1330.52	1155.49	1100.13	1996.95	1991.27	2111.47
CALB2	232.14	225.58	215.05	1048.84	1097.65	667.14
CALM1	3181.60	2794.74	2954.73	5478.61	5652.11	5152.85
CAMK2D	1752.24	1939.13	1892.63	3499.27	3516.77	3916.27
CAMK2G	389.43	421.78	451.32	666.83	734.87	734.72
CAST	186.34	195.40	220.23	369.93	394.14	419.32
CCL5	35.98	23.58	29.14	13.65	15.75	2.50
CCND1	1241.51	1276.23	1254.19	744.03	772.53	659.63
CD44	10.91	13.13	9.89	71.05	67.18	41.34
CDK2	126.71	150.12	112.84	65.11	49.73	11.30
CDS1	79.18	103.68	108.39	229.40	265.56	146.47
CHL1	1469.65	1528.15	1613.41	3029.18	3072.26	2454.41
CHMP2B	661.64	676.02	720.17	989.46	1056.32	1112.70
CHRNA7	404.98	370.70	389.10	777.67	811.10	814.82
CLU	2347.66	2072.63	2170.38	7786.52	7881.12	7783.69
CNR1	627.94	640.03	609.82	1373.46	1217.96	1548.26
CNTN1	1816.19	1904.30	1876.34	3552.71	3769.34	3430.65
CNTNAP1	576.09	681.83	623.15	907.32	989.27	1245.37
COL4A1	274.49	272.02	250.60	838.04	771.61	554.49
CPLX1	284.86	283.63	296.52	560.94	542.92	496.92
CRH	76.59	51.44	52.84	169.03	238.92	429.33
CSF1	50.67	43.31	45.44	105.69	75.44	73.88
CX3CL1	64.49	68.85	69.88	161.11	143.41	123.95
CXCR4	513.00	407.85	466.87	816.27	788.14	742.23

**CYP4X1**	273.63	301.04	314.30	552.03	604.45	554.49
**DAGLA**	336.71	366.05	300.22	546.09	552.10	539.47
**DGKB**	681.52	785.15	787.57	1757.45	1658.80	1853.65
**DLGAP1**	347.08	391.59	403.17	981.55	1102.24	862.39
**DRD2**	149.18	143.15	146.91	259.09	254.54	199.04
**EFR3A**	410.17	403.20	409.10	708.40	706.40	764.76
**EGFR**	96.47	104.84	105.43	197.73	225.15	181.52
**EPHA4**	1931.13	2139.97	2007.43	1246.78	1242.76	1307.95
**EPHA5**	175.97	189.59	179.50	734.13	682.52	584.53
**EPHA6**	93.01	116.45	126.17	239.29	239.84	184.02
**ERLEC1**	1751.38	1732.48	1730.43	2614.50	2673.66	2654.67
**FGF12**	550.16	486.79	561.67	1485.29	1434.71	1568.28
**FOS**	500.91	539.03	546.86	983.53	928.66	1060.14
**FRMPD4**	177.70	179.14	189.86	267.99	312.40	259.12
**GAA**	240.79	261.57	260.97	361.02	405.16	436.84
**GABRA1**	234.74	268.53	267.63	590.63	591.60	549.49
**GABRA4**	144.86	152.44	180.24	483.74	558.53	544.48
**GABRB2**	831.02	912.86	988.29	1643.64	1734.11	1525.73
**GABRG2**	361.77	346.32	359.48	799.45	797.32	652.12
**GAD1**	3793.44	4288.86	4409.38	10285.44	10289.23	11766.26
**GAL3ST1**	10.91	9.65	19.51	47.30	39.62	31.33
**GBA**	246.84	232.55	260.23	564.90	636.60	516.95
**GDPD2**	136.22	167.53	140.24	677.72	690.79	579.53
**GLRB**	428.31	458.93	423.91	971.65	1092.14	929.97
**GLS**	1412.62	1553.69	1632.66	2414.59	2432.12	2381.82
**GNAO1**	2888.64	2923.60	3047.32	4846.21	5040.44	4461.97
**GNPTAB**	1260.52	1271.59	1321.59	1874.23	1987.60	1986.32
**GNPTG**	410.17	350.96	346.88	759.86	800.08	732.22
**GPD1L**	290.91	277.82	331.33	646.05	684.36	602.05
**GRIA1**	1471.38	1467.78	1507.49	2608.57	2769.18	2849.91
**GRIA2**	2817.78	3314.84	3131.01	5621.12	5630.98	7002.70
**GRIA4**	2086.68	2245.61	2225.93	3307.27	3422.18	3498.24
**GRIK2**	520.78	532.07	590.56	897.42	866.20	889.92
**GRIN1**	86.10	100.20	63.21	270.96	266.47	246.60
**GRIN2B**	897.57	990.64	1041.62	1718.85	1725.85	1778.55
**GRM5**	446.46	533.23	526.86	1282.41	1285.01	1232.86
**GRN**	460.29	375.34	345.40	743.04	766.10	734.72
**GUCY1B3**	281.40	244.16	264.67	672.77	637.52	469.38
**HAP1**	290.05	313.81	318.74	495.62	561.29	589.54
**HCN1**	55.85	54.92	65.43	262.06	267.39	246.60
**HDAC1**	966.70	908.21	930.52	596.56	549.35	567.01
**HEXB**	408.44	395.08	406.88	683.66	731.20	649.61
**HMOX1**	23.88	13.13	9.14	90.84	63.50	101.42
**HSPB1**	305.60	249.96	260.23	610.42	523.63	559.50
**IL13RA1**	78.32	47.96	58.77	91.83	95.65	96.41
**INHBB**	10.05	6.16	13.59	55.21	62.58	31.33
**INPP5F**	2608.65	2577.64	2563.67	4177.19	4362.64	4251.70
**ITPR1**	29.06	40.99	41.73	114.59	124.12	61.37
**ITPR2**	20.42	17.77	18.77	216.53	215.96	229.08
**LAMB2**	103.38	96.72	110.61	208.61	204.02	153.98

**LAMP1**	1212.13	1102.09	1031.99	1675.31	1888.41	1871.17
**LIF**	28.20	29.38	22.48	10.68	19.42	2.50
**LPAR1**	93.88	75.82	66.92	257.11	226.98	138.96
**MAGEE1**	652.14	662.09	673.51	1156.72	1185.82	1190.30
**MAP2K1**	655.59	672.54	652.77	1194.33	1261.13	1117.71
**MAPT**	2078.90	1942.61	2055.58	3575.47	3445.14	3355.56
**MYC**	226.96	222.10	196.53	374.88	355.56	379.27
**NEFH**	61.04	57.24	66.92	163.09	172.80	158.99
**NEFL**	12334.15	11433.28	11307.85	21757.71	23144.39	22542.47
**NES**	6665.98	6645.57	5963.28	3727.88	3473.61	3308.00
**NGF**	4.86	6.16	3.22	1.77	1.00	2.50
**NLGN4X**	1399.66	1459.66	1403.80	2152.33	2073.93	2281.69
**NMB**	169.06	144.31	156.54	306.59	302.29	244.10
**NOL3**	105.11	101.36	99.50	186.84	207.70	219.07
**NOTCH3**	528.56	455.45	432.06	352.12	322.50	264.12
**NPAS4**	25.61	21.25	29.88	118.55	156.26	96.41
**NPC2**	799.91	706.21	767.58	1584.26	1511.86	1598.32
**NPTN**	2174.83	2156.22	2248.89	3537.87	3698.62	3668.46
**NPY**	2.27	7.32	3.96	808.35	710.07	409.31
**NQO1**	112.89	117.61	98.02	448.11	437.30	441.85
**NRXN1**	780.90	696.92	656.48	1506.07	1436.55	2016.35
**NSF**	1613.97	1511.90	1502.31	2323.54	2430.28	2306.72
**NTNG1**	104.25	172.18	159.50	438.22	395.97	554.49
**NTRK1**	773.12	763.09	676.48	225.44	193.00	133.96
**NTS**	739.42	737.55	886.08	2647.16	3122.77	3060.18
**OLFM3**	468.93	454.28	445.39	1006.29	992.95	827.34
**OLIG2**	383.38	400.88	338.74	240.28	210.45	161.49
**OPTN**	1138.67	1158.97	1229.01	1926.68	1990.35	2001.33
**PCNA**	548.44	471.70	439.47	248.20	248.11	234.09
**PDGFRB**	26.47	25.90	18.03	322.43	271.07	174.01
**PIK3CB**	120.67	94.39	101.73	250.18	236.17	204.05
**PINK1**	788.68	757.29	763.13	1417.00	1386.95	1207.82
**PLA2G16**	192.39	190.75	203.20	739.08	775.28	722.21
**PLA2G4C**	137.08	159.41	170.61	306.59	317.91	254.11
**PLCB1**	252.02	299.88	291.33	582.71	597.11	511.94
**PLXNB3**	15.24	28.22	18.03	82.93	67.18	63.87
**PMP22**	76.59	46.80	40.25	264.04	239.84	151.48
**PPARGC1A**	297.82	316.13	335.77	587.66	651.29	654.62
**PPT1**	1057.44	923.30	994.96	1733.70	1743.30	1718.47
**PRKCA**	1229.41	1301.77	1363.06	2106.80	2136.38	2048.90
**PRKCE**	199.31	207.00	193.57	373.89	362.91	286.65
**PRNP**	2351.12	2102.82	2285.18	4677.97	4904.51	4619.67
**PTDSS1**	1184.47	1104.41	1123.09	1726.77	1786.46	1640.88
**PTPRR**	77.46	88.59	93.58	269.97	271.99	296.67
**RIMS1**	410.17	362.57	377.99	825.18	816.61	804.81
**RTN4**	6812.89	6659.50	6788.37	10387.38	10513.32	10061.59
**RYR1**	46.35	92.07	85.43	137.36	124.12	171.51
**RYR2**	164.74	253.44	208.38	583.70	557.61	554.49
**RYR3**	245.11	254.60	283.93	423.37	449.24	409.31
**S100B**	58.44	37.51	46.18	1005.30	857.02	514.44

**SCN1A**	473.25	520.46	485.39	1278.45	1296.95	1355.51
**SCN2A**	1780.76	1879.92	1914.11	3230.08	3262.37	3202.86
**SHH**	608.93	664.41	607.59	305.60	268.31	404.30
**SIRT2**	1148.18	1088.16	1147.53	1915.80	1950.86	1861.16
**SLC1A1**	594.24	573.86	594.26	1019.15	1059.99	1087.67
**SLC2A1**	240.79	244.16	228.38	126.47	128.71	133.96
**SLC32A1**	341.90	280.14	326.89	654.96	623.74	737.23
**SLC4A10**	313.38	419.46	368.36	557.97	584.25	587.03
**SLC9A6**	801.64	799.08	824.61	1267.56	1347.46	1260.39
**SNAP91**	1585.45	1695.33	1611.18	2724.36	2748.97	3092.72
**SNCA**	544.98	551.80	506.87	1044.89	1180.31	1127.72
**SNCB**	332.39	337.03	331.33	940.97	946.11	909.95
**SORCS3**	210.54	245.32	210.60	377.85	405.16	461.88
**SORL1**	197.58	207.00	231.34	500.57	499.75	511.94
**SP1**	184.61	182.63	173.57	131.42	121.36	93.91
**STX1A**	636.58	628.43	649.07	976.60	998.46	1087.67
**SYT1**	6142.29	5704.05	5707.01	9777.74	9523.26	9578.48
**SYT13**	1792.86	1756.86	1775.61	3231.07	3175.12	3130.27
**SYT4**	1582.00	1482.88	1452.68	3110.33	3210.02	3115.25
**SYT7**	520.78	523.94	575.75	814.29	849.67	782.28
**TBR1**	144.00	155.92	166.90	54.23	61.67	36.33
**TGFB1**	84.37	93.23	89.14	167.05	215.04	148.98
**TH**	277.08	309.17	323.18	563.91	621.90	572.02
**THY1**	1422.12	1289.00	1400.84	3422.08	3560.86	3458.19
**TLR4**	3.14	1.16	1.00	64.12	67.18	51.35
**TNC**	90.42	103.68	94.32	578.75	612.72	662.13
**TNFRSF10B**	503.50	570.38	500.20	326.38	348.21	304.17
**TNFRSF1A**	179.43	169.85	146.17	281.85	298.62	264.12
**TNR**	455.97	442.68	479.46	1096.35	1130.71	1092.68
**TP53**	923.49	932.59	906.08	400.61	416.18	321.70
**TSPO**	1.00	1.16	1.00	2.76	2.89	2.50
**UNC13A**	1225.09	1403.93	1460.83	1966.27	2051.89	2176.56
**WFS1**	114.62	111.81	113.58	204.66	228.82	229.08
**XK**	115.48	94.39	117.28	206.63	183.82	169.00

Supplementary Figure S5.

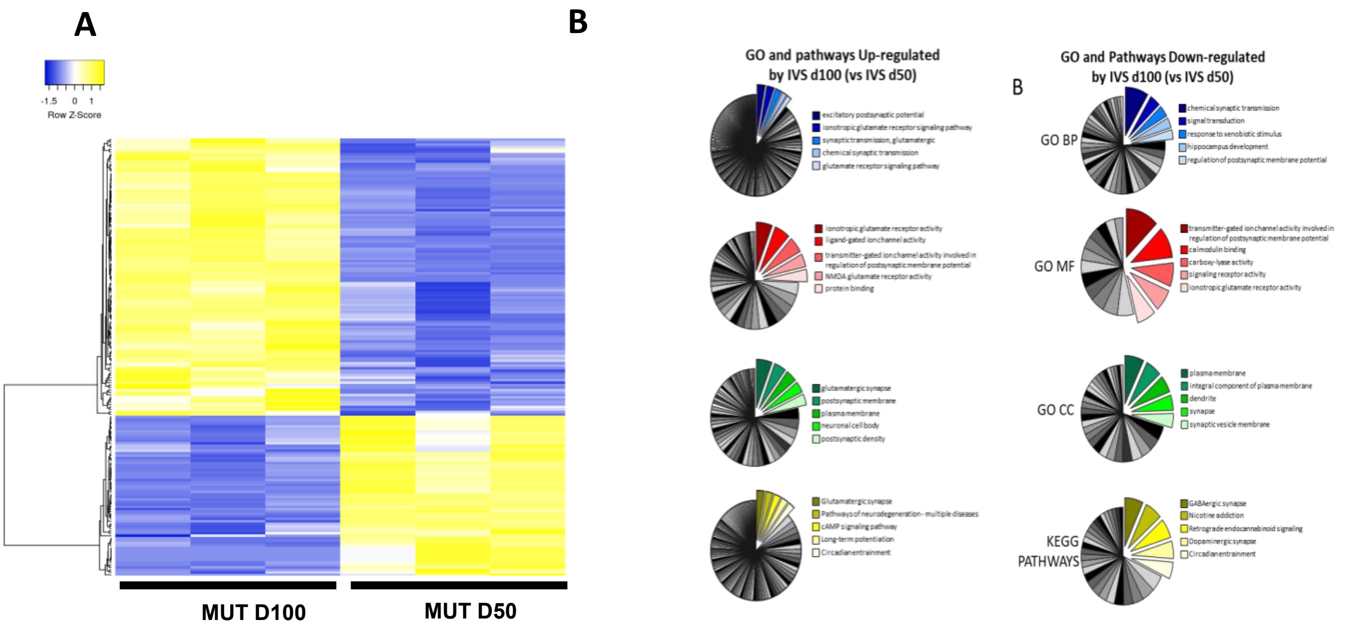

Figure S5. A) Heat map of unsupervised hierarchical clustering of 180 differentially expressed (DE) genes between D50 MUT and D100 MUT (114 genes up- and 66 genes down-regulated in D100 MUT). Average linkege and Pearson Distance Measurement Methods wereperformed for Hierarchical cluster using Heatmapper tools (http://www.heatmapper.ca). The significant DE genes are selected by multiple t-test performed with Graph Pad Prism 6, with p-value ≤ 0.05 and a fold change cut-off values of ≥1.5 or ≤0.66 for upmodulated and downregulated genes respectively. **B)** Gene Ontology (GO) and KEGG Pathways enrichment analysis of 180 down- and up-regulated genes by D100 MUT (respect to D50 MUT) have been analyzed by DAVID database. An overview of top 5 significantly enriched terms in KEGG pathways and three GO categories: biological process (BP), cellular component (CC), and molecular function (MF). The cut-off of p-value was set to 0.05 and are selected only enriched terms in which are represented a minimum of 3 genes. The −log10 p-value associated with each category is represented by the size of the pie slice.

Supplementary Table S8

Probe Name	IVS d50 _1	IVS d50 _2	IVS d50 _3	IVS d100 _	IVS d100	IVS d100 _3
ACAA1	531.82	431.67	430.42	787.08	773.70	847.09
ACHE	695.36	730.74	684.34	350.59	303.97	488.95
ACIN1	2713.22	2282.48	2581.41	4091.90	3976.09	4462.21
ACTN1	546.04	514.02	523.80	844.56	708.91	847.09
ADORA1	135.08	143.43	193.47	224.91	275.39	265.12
ADORA2A	31.27	15.56	49.15	85.58	85.78	94.50
ADRA2A	103.79	19.90	59.95	358.39	644.12	879.18
ALDH1L1	1.42	2.17	2.07	15.43	18.13	8.34
AMPH	3439.88	2848.12	3261.37	1470.06	1265.35	1566.73
AP3M2	918.62	700.40	869.57	1269.36	1302.51	1254.21
APC	1645.27	1190.20	1453.82	2193.96	2240.07	2365.77
AQP4	1.42	2.17	1.00	10.56	5.74	4.97
ATP6V0E1	1397.84	1144.68	1199.13	2150.12	1911.36	2152.08
BAX	1577.02	1565.12	1301.77	2385.90	2127.64	2199.38
BCHE	126.54	214.95	175.72	613.65	762.27	529.50
BID	167.78	165.10	146.39	411.97	423.07	459.39
C3	1.42	2.17	2.07	1.00	1.00	1.00
CACNA1C	1719.22	1196.70	1564.19	723.75	670.80	968.72
CACNB2	337.00	323.31	421.15	208.34	162.00	287.92
CADPS	1945.32	1242.21	1574.99	895.22	793.71	1002.50
CALB2	261.64	290.80	269.88	1107.62	863.27	505.85
CALM1	3486.81	3194.88	3096.98	4470.90	5132.80	5099.92
CASP6	423.75	364.48	376.39	804.61	867.08	772.76
CASP7	96.68	63.24	73.84	198.60	157.24	134.20
CAST	472.10	329.81	404.17	255.11	181.06	293.84
CCND1	1474.63	1456.76	1273.99	282.39	121.03	285.39
CDK5	453.61	429.50	417.29	656.52	646.03	694.20
CLDN15	2.83	24.23	7.47	42.71	36.23	27.77
CLN3	76.77	54.57	68.44	107.99	113.41	118.15
CLU	5213.15	3279.40	3802.40	8411.94	5885.52	9574.90
CNKSR2	1594.08	1296.39	1446.10	2877.92	2923.24	2627.62
CNTF	126.54	93.58	137.13	215.16	249.66	238.93
CRH	592.97	557.37	525.35	49.53	53.38	141.80
CRTC2	226.09	178.10	223.57	318.44	312.54	310.73
CTNS	28.42	6.89	27.54	60.25	47.66	70.85
CUL1	1962.39	1955.22	1955.49	2995.81	3038.53	2783.88
CUL3	1595.50	1532.62	1443.79	2236.83	2461.13	2235.70
CXXC1	132.23	93.58	126.33	215.16	204.88	221.20
CYP4X1	464.99	238.79	382.56	85.58	61.00	150.25
DAGLA	324.21	282.13	354.01	474.33	471.66	510.91
DDC	17.05	19.90	25.99	1.00	4.79	1.00
DDIT3	1124.81	1580.30	1243.12	778.31	572.66	925.64
DDX23	568.80	581.21	531.52	873.79	835.64	874.11
DGKB	351.22	178.10	252.90	555.19	470.71	418.85
DLGAP1	551.73	579.04	658.87	1927.98	2297.24	2162.21
DLX1	1080.73	1504.44	1239.26	420.74	139.13	84.36
DLX2	543.20	973.47	746.85	203.47	87.68	34.53
DRD4	73.93	41.57	70.76	34.92	23.84	25.24
EFNA1	34.11	17.73	43.74	68.04	101.97	128.28

**EGF**	39.80	37.23	56.09	79.74	111.50	84.36
**EGR1**	18.47	13.39	40.66	57.33	68.63	108.01
**EPHA3**	3512.40	2360.50	3264.46	580.53	581.24	664.64
**EPHA5**	274.43	253.96	270.65	1443.75	1858.95	1322.63
**EPHA7**	861.73	804.43	935.94	3392.35	3918.92	3207.05
**FA2H**	1.42	2.17	2.07	1.00	1.00	1.00
**FAM126A**	573.06	479.35	481.35	1067.68	1162.45	1331.92
**FGF12**	413.79	297.30	369.44	531.81	549.79	751.64
**FGF14**	2791.43	1697.33	2325.18	456.79	263.95	606.36
**GABRA1**	129.39	119.59	154.11	60.25	35.28	61.56
**GABRB2**	725.22	676.56	706.72	296.03	289.68	390.97
**GAD1**	1353.76	1380.91	1370.46	436.33	132.46	336.91
**GAD2**	432.28	581.21	522.26	95.33	25.75	33.68
**GALC**	543.20	412.16	471.32	253.16	278.24	249.07
**GBA**	351.22	217.11	314.65	549.35	483.10	634.23
**GDPD2**	446.50	214.95	330.85	685.75	628.88	710.25
**GLRB**	614.30	561.70	620.28	1435.96	1599.79	1523.65
**GNAI1**	1386.46	1181.53	1294.06	1891.93	2240.07	2277.09
**GNGT1**	1.42	2.17	2.84	1.00	1.00	1.00
**GPD1L**	305.72	344.98	314.65	899.12	1050.97	1041.36
**GRIA1**	1548.58	1378.74	1491.64	2941.25	4038.98	3035.58
**GRIA2**	10043.80	9713.88	11036.46	5869.02	5573.95	6001.17
**GRIA3**	1035.22	839.11	953.69	5194.81	6316.19	6420.96
**GRIA4**	837.56	622.38	818.63	424.64	449.75	489.80
**GRIK2**	699.62	503.19	682.79	910.81	1147.20	1067.54
**GRIN1**	32.69	41.57	64.58	188.86	172.48	204.30
**GRIN2A**	39.80	32.90	53.01	105.07	85.78	79.29
**GRIN2B**	1130.50	1008.15	1193.72	1739.94	2213.40	2027.91
**GRIN2C**	1.42	2.17	6.70	47.58	47.66	63.25
**GRIN2D**	514.76	592.04	557.76	206.40	198.21	241.47
**GRIN3B**	4.25	2.17	1.00	7.64	7.65	5.81
**HGF**	1.42	2.17	5.15	81.69	88.64	68.31
**HIF1A**	2526.93	2256.47	2239.51	3784.02	3835.07	3627.69
**HNRNPM**	5281.41	4161.46	4463.06	8462.60	8759.18	7589.96
**HSPB1**	292.92	169.43	195.02	493.81	478.33	494.86
**IDE**	261.64	243.12	313.10	512.33	477.38	526.12
**IDH1**	1511.60	1333.23	1383.58	2374.21	2599.28	2459.53
**IGF1R**	2170.00	1849.03	2010.28	1249.87	1255.82	1174.81
**IKBKB**	253.10	236.62	238.24	482.12	474.52	442.50
**INHBB**	34.11	28.56	46.83	179.11	153.43	121.53
**ITPR2**	36.95	2.17	33.71	153.78	100.07	112.24
**KATNA1**	501.96	511.86	513.77	834.82	813.72	744.04
**KCNB1**	437.97	412.16	424.24	232.70	234.41	270.19
**KCNJ10**	1.42	2.17	4.38	74.86	61.00	66.63
**KIAA1161**	534.67	546.53	540.01	880.61	1077.65	868.20
**LAMB2**	184.85	110.92	162.60	319.41	267.76	332.69
**LAMP1**	1130.50	841.27	927.45	1541.18	1532.14	1542.24
**LIF**	4.25	6.89	9.79	43.69	21.94	52.27
**LRRC25**	1.42	2.17	2.07	1.00	1.00	1.00
**MAPK10**	3569.28	2655.24	3090.80	1827.63	1837.99	2271.17

**MBP**	38.38	43.74	49.92	7.64	1.00	19.32
**MGMT**	69.66	37.23	62.27	34.92	24.80	16.79
**MSN**	516.18	475.01	481.35	1072.55	1079.56	918.04
**MTA2**	1743.39	1842.53	1696.16	2946.12	3104.27	2697.72
**MTOR**	564.53	457.67	547.73	773.44	811.82	798.10
**MYD88**	1.42	2.17	15.96	41.74	37.18	38.75
**NAGLU**	92.41	76.24	86.19	154.76	138.18	172.21
**NEFH**	1.42	2.17	19.05	28.10	24.80	27.77
**NEFL**	5437.84	5942.91	5513.47	1932.85	1332.05	2682.52
**NELL2**	2522.67	1597.63	1842.80	9024.77	10758.17	10236.26
**NGFR**	103.79	76.24	97.00	13.48	18.13	29.46
**NLGN4X**	2652.07	2488.36	2589.90	1752.61	1583.59	1548.99
**NMB**	152.14	95.75	125.56	236.60	256.33	265.12
**NOSTRIN**	1.42	2.17	5.15	20.30	19.08	15.10
**NOVA1**	5096.55	3816.87	4672.99	1846.14	1668.39	1721.30
**NPAS4**	1.42	2.17	21.36	56.35	40.99	104.63
**NPY**	8.51	11.23	19.05	142.09	171.53	190.79
**NTNG1**	1176.00	787.09	1072.55	334.03	227.75	451.79
**NTS**	342.69	442.50	379.48	2246.58	1618.84	2675.76
**P2RX4**	5.67	4.73	20.59	23.23	28.61	26.93
**PARP1**	2625.05	2304.15	2256.49	3691.46	3710.26	3384.43
**PCSK2**	2472.90	1768.84	2308.20	453.87	302.06	600.45
**PDE1B**	156.41	126.09	167.23	30.05	4.79	54.80
**PDE4D**	312.83	210.61	295.35	402.23	516.45	554.84
**PDGFRB**	180.58	104.42	146.39	515.25	350.66	554.84
**PECAM1**	1.42	2.17	2.07	1.00	1.00	1.00
**PHF19**	36.95	56.74	62.27	145.01	162.95	116.46
**PIK3CB**	152.14	104.42	174.18	317.47	340.18	284.55
**PLCB1**	574.48	433.84	519.94	892.30	953.78	808.23
**PLCB3**	152.14	132.59	146.39	230.75	247.75	236.40
**PLXNC1**	1666.60	1688.66	1710.83	602.93	588.86	522.74
**PMP22**	68.24	48.07	62.27	159.63	209.64	199.24
**POLR2B**	2235.42	2113.43	2158.47	3394.30	3531.13	3228.17
**POLR2J**	119.43	139.09	153.34	188.86	218.22	216.13
**PPM1L**	628.52	587.71	614.10	889.38	1139.58	991.52
**PRKCA**	1046.60	1079.67	1135.07	2464.82	2360.13	2303.27
**PRKCB**	544.62	509.69	537.69	221.01	414.50	335.23
**PSMB8**	1.42	2.17	1.00	9.59	14.32	11.72
**PTPRN2**	2403.22	1944.39	2001.79	963.43	900.43	1059.09
**PTPRR**	184.85	152.10	174.95	34.92	20.03	34.53
**RAB3C**	2707.53	2273.81	2308.20	722.77	561.23	650.28
**RAF1**	1844.36	1606.30	1710.83	2637.27	2832.72	2678.30
**RAPGEF2**	995.41	912.79	928.22	1536.31	1564.53	1556.59
**RASGRP1**	947.06	615.88	801.65	258.03	269.67	349.58
**RET**	179.16	71.91	141.76	32.00	4.79	43.82
**RYR1**	95.26	95.75	130.19	49.53	34.33	59.02
**RYR2**	275.86	260.46	273.74	829.95	766.08	755.86
**RYR3**	226.09	156.43	217.40	83.63	52.43	107.17
**SCAMP2**	474.94	338.48	354.78	646.78	625.07	633.39
**SF3A2**	1110.59	1465.43	1235.40	822.15	778.47	852.15

**SIRT7**	349.80	312.47	295.35	578.58	532.64	688.29
**SLA**	32.69	24.23	47.60	897.17	1312.04	1253.36
**SLC12A5**	698.20	698.24	657.32	155.73	142.95	196.70
**SLC17A6**	3018.96	2362.66	2693.33	451.92	290.63	744.88
**SLC18A3**	1.42	2.17	2.07	1.00	1.00	1.00
**SLC32A1**	115.17	152.10	133.27	21.28	1.00	3.28
**SLC8A1**	4715.44	4040.09	4384.33	2752.24	2854.64	2818.51
**SNCA**	683.98	449.01	509.14	1382.37	1493.07	1411.31
**SNCAIP**	335.58	388.32	360.18	633.14	664.13	492.33
**SORCS3**	248.84	201.94	258.30	69.02	59.10	121.53
**SOX9**	2828.41	2616.23	2515.81	4506.95	4188.57	3614.17
**STAMBPL1**	1542.89	1159.85	1358.89	310.65	296.35	416.31
**STX1A**	557.42	509.69	523.80	836.77	868.98	1046.42
**SYT1**	4881.82	3327.08	4087.19	5869.99	7145.13	6863.56
**SYT13**	2583.82	2670.41	2492.66	1305.40	1548.34	1702.72
**SYT4**	1975.19	1627.97	1754.82	3294.92	3464.43	4843.99
**TAF6L**	174.89	132.59	157.20	261.93	255.38	272.72
**TCIRG1**	2.83	2.17	27.54	36.87	47.66	60.71
**TENM2**	581.59	305.97	522.26	94.35	55.29	103.79
**TF**	39.80	24.23	42.20	19.33	13.36	13.41
**TGFB1**	51.18	115.25	86.97	221.98	208.69	139.27
**TH**	110.90	212.78	189.61	27.12	10.50	45.51
**TNC**	244.57	321.14	282.23	741.29	556.46	684.07
**TP53**	1379.35	1315.89	1246.21	818.25	822.30	661.26
**TRIM37**	826.18	776.26	721.38	1278.12	1301.56	1300.66
**TSPO**	19.89	28.56	27.54	62.20	100.07	68.31
**UBE2N**	1.42	2.17	2.07	1.00	1.00	1.00
**UGT8**	140.76	178.10	200.42	76.81	68.63	81.83
**VCP**	2064.77	1753.67	1909.18	3034.78	3165.25	3007.71
**WFS1**	133.65	74.08	102.40	198.60	162.95	197.55
**XBP1**	449.34	468.51	387.97	217.11	217.26	238.09

Supplementary Figure S6.

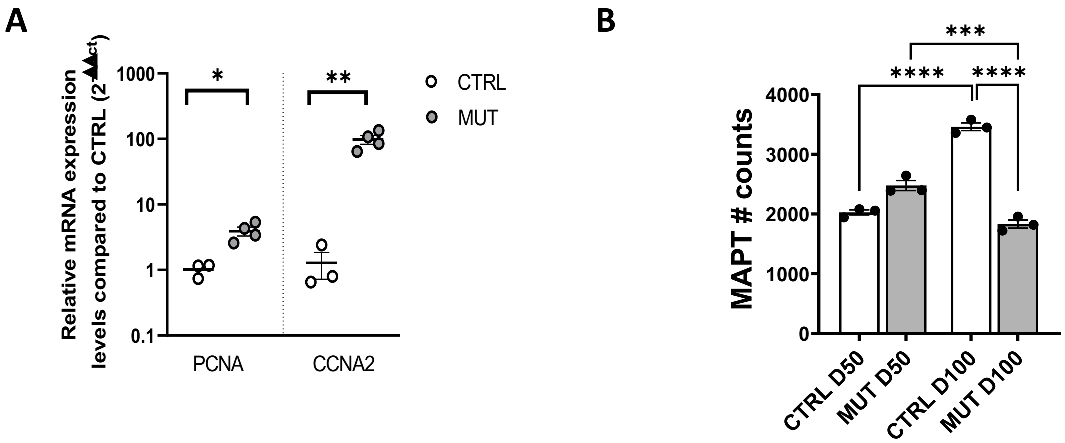

Figure S6. A) Scatter Dot plot reporting the Real-time PCR analysis of PCNA and CCNA2 on control (CTRL) and tau-mutant (MUT) derived cortical organoids at D100. (CTRL n= 3/2, MUT n=4/2; replicates/batches). Gene expression is normalized to the housekeeping gene ATP5O (*$p<0.05$, MW test; ** $p<0.001$ MW test); **B)** Scatter Dot plot reporting the number of MAPT normalized counts on control (CTRL) and tau-mutant (MUT) derived cortical organoids at D50 and D100. (CTRL n= 3 batches, MUT n=3; batches, One-way ANOVA with Holm-Sidak multiple comparisons).

Supplementary Figure S7.

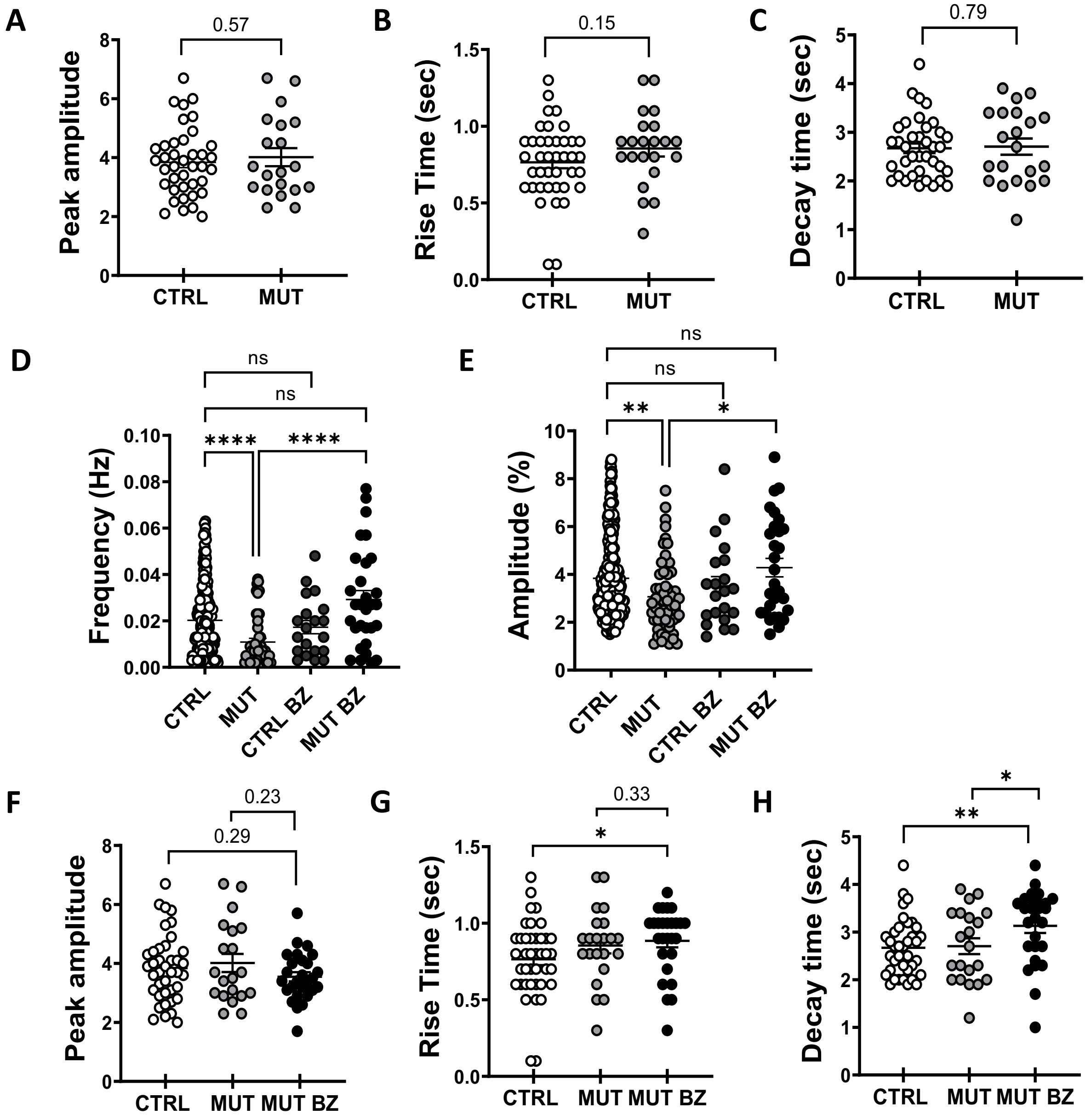

Figure S7. Scatter dot plots showing the kinetics parameters of spontaneous calcium oscillations in day 100 (D100) control (CTRL, white dots) and mutant (MUT, grey dots) samples. **A–C:** Organoids – **A)** Peak amplitude, **B)** Rise time, **C)** Decay time (CTRL: n = 47/4; MUT: n = 29/4; fields of view (FOVs)/batches; Mann–Whitney test). **D–E:** Dissociated neurons – **D)** Transient frequency, **E)** Amplitude in untreated MUT (grey dots) and BZ-treated MUT (black dots) (CTRL: n = 282/3; MUT: n = 55/3; CTRL BZ: n = 23/3; MUT BZ: n = 30/3; FOVs/batches; Kruskal–Wallis test with Dunn's multiple comparisons). **F–H:** Organoids – **F)** Peak amplitude, **G)** Rise time, **H)** Decay time in CTRL, untreated MUT, and BZ-treated (black dots) MUT organoids (CTRL: n = 47/4; MUT: n = 29/4; MUT BZ: n = 29/4; FOVs/batches; Kruskal–Wallis test with Dunn's multiple comparisons).

Supplementary Figure S8.

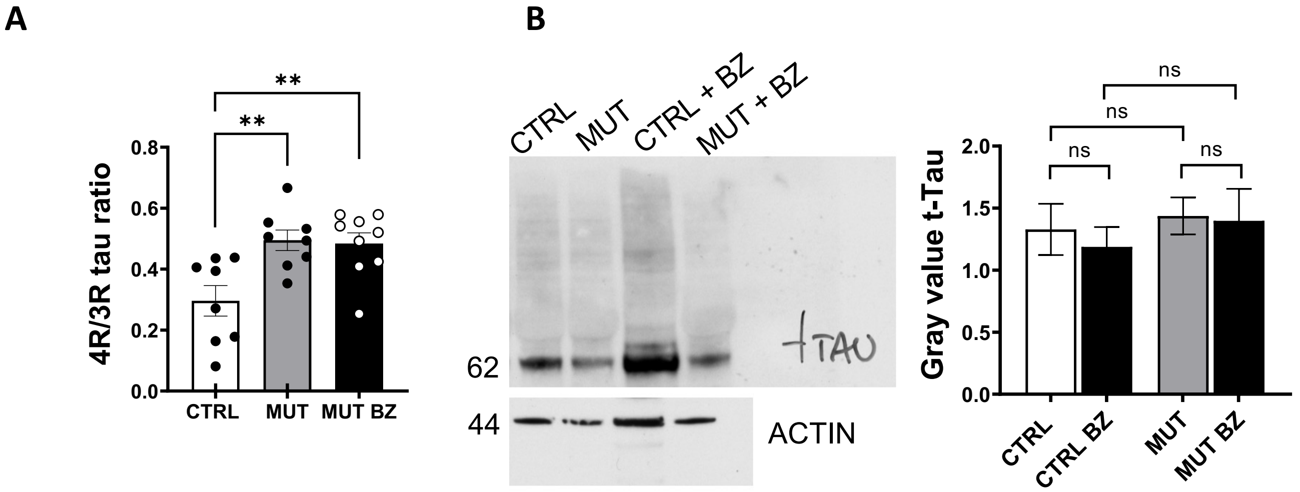

Figure S8. A) Scatter dot plot showing the RT-qPCR analysis of the 4R/3R tau ratio in CTRL (white bars), MUT (grey bars) and MUT BZ (black bars) cortical organoids at D100 (CTRL n = 4/2; MUT n= 4/2; MUT BZ n=4/2; batches/replicates, 10 organoids each). Gene expression is normalized to the housekeeping gene ATP5O (** p<0,01, one–way ANOVA with Tukey test for multiple comparison); **B)** Representative immunoblot of t-tau. Bar graph showing the amount of t-tau protein in D100 untreated and BZ treated CTRL and MUT organoids. Actin was used as a protein loading control. Values are expressed as median ± sem from 3 independent experiments (batches); One-Way ANOVA with Tukey's multiple comparisons.
